# Translin facilitates RNA polymerase II dissociation and suppresses genome instability during RNase H2- and Dicer-deficiency

**DOI:** 10.1371/journal.pgen.1010267

**Published:** 2022-06-17

**Authors:** Natalia Gomez-Escobar, Ahad A. A. Alsaiari, Hanadi A. S. Alahamadi, Othman Alzahrani, Ellen Vernon, Hussam A. E. Althagafi, Nasser S. Almobadel, David W. Pryce, Jane A. Wakeman, Ramsay J. McFarlane

**Affiliations:** North West Cancer Research Institute, School of Medical Sciences, Bangor University, Bangor, United Kingdom; Lerner Research Institute, UNITED STATES

## Abstract

The conserved nucleic acid binding protein Translin contributes to numerous facets of mammalian biology and genetic diseases. It was first identified as a binder of cancer-associated chromosomal translocation breakpoint junctions leading to the suggestion that it was involved in genetic recombination. With a paralogous partner protein, Trax, Translin has subsequently been found to form a hetero-octomeric RNase complex that drives some of its functions, including passenger strand removal in RNA interference (RNAi). The Translin-Trax complex also degrades the precursors to tumour suppressing microRNAs in cancers deficient for the RNase III Dicer. This oncogenic activity has resulted in the Translin-Trax complex being explored as a therapeutic target. Additionally, Translin and Trax have been implicated in a wider range of biological functions ranging from sleep regulation to telomere transcript control. Here we reveal a Trax- and RNAi-independent function for Translin in dissociating RNA polymerase II from its genomic template, with loss of Translin function resulting in increased transcription-associated recombination and elevated genome instability. This provides genetic insight into the longstanding question of how Translin might influence chromosomal rearrangements in human genetic diseases and provides important functional understanding of an oncological therapeutic target.

## Author summary

Human genetic diseases, including cancers, are frequently driven by substantial changes to chromosomes, including translocations, where one arm of a chromosome is exchanged for another. The human nucleic acid binding protein Translin was first identified by its ability to bind to the chromosomal sites at which some of these translocations occur. This resulted in Translin being implicated in the mechanism that generated the translocation and thus the associated disease state. However, since its discovery there has been little evidence to directly indicate Translin does contribute to this process. It is, however, known to contribute to a number of biological functions including, amongst others, neurological regulation, sleep control, vascular stiffening, cancer immunomodulation and it has been recently identified as a potential therapeutic target in some cancers. Here we demonstrate that Translin has conserved function in genome stability maintenance when other primary pathways are defective, a function independent of a key binding partner protein, Trax. Specifically, we demonstrate that Translin contributes to minimizing the deleterious genome destabilizing effects of retaining gene expression machineries on chromosomes. This offers the first evidence for how Translin might contribute to genetic disease-causing chromosomal changes and offers insight to inform therapeutic design.

## Introduction

Genetic diseases and evolution can be driven by large scale structural changes to chromosomes, including chromosomal translocations [1–3]. Translin is a conserved nucleic acid binding protein first identified in humans by its ability to bind to malignant disease-associated chromosomal translocation breakpoint junctions, which resulted in the postulate that it mediates genetic recombination [4]. Subsequently, it was found to form a hetero-octamer (also known as C3PO) with a paralogous protein, Translin-associated factor X (Trax) [5,6]. Translin and the Translin-Trax (Tn-Tx) complex can bind to both RNA and DNA, and the Tn-Tx complex possesses endoribonuclease activity, which facilitates passenger strand removal from small interfering RNAs during RNA interference (RNAi) in higher eukaryotes and can process other RNAs, including tRNA precursors [7–11]. Translin and Trax (individually or in a Tn-Tx complex) are implicated in an array of biological processes, many of which influence human neurological function and disease, including cancer [5,12,13].

Not all the functions of Translin and Trax require endoribonuclease activity; however, the RNase activity causes premature degradation of precursor-miRNAs (pre-miRNAs) of tumour suppressor miRNAs in cancers that have insufficiency in the RNase III Dicer, which, when at normal levels processes pre-miRNA to mature tumour suppressing miRNAs. Inhibition of Tn-Tx in Dicer-limited cancer cells permits the residual Dicer to re-establish appropriate maturation of pre-miRNAs [14]. Indeed, small molecule inhibitors of Tn-Tx RNase activity have been developed that enable pre-miRNA maturation by Dicer, demonstrating the therapeutic potential of targeting Tn-Tx in Dicer-limited cancers [15].

The function of Translin in other processes also indicates that regulating its activity might be of additional clinical utility, for example, deletion of *Tsn* (Translin gene) in mice reduces hypertension-related vascular stiffening by maintaining levels of a regulatory miRNA [16,17]. Therapeutically targeting a specific function of a complex whose constituent parts, together or individually, act in a diverse range of processes requires an understanding of all functional roles to ensure disease-specific targeting. For example, murine Translin is also implicated in survival of T cells required for immunological tumour suppression, so oncological therapeutic targeting needs to avoid diminution of such beneficial activities [18].

Despite the original suggestion that Translin functions in recombination control [4,19], there is currently only very limited evidence to support any involvement in genome stability regulation. For example, Translin-deficient mice exhibit delayed proliferation in haemopoietic stem cells following ionizing irradiation [20], Translin migrates to the nucleus following DNA damage [20], Translin interacts with the DNA damage-inducible GADD34 [21] and recently Translin has been shown to specifically bind to short open reading frame-encoded peptides following UV irradiation [22]. Trax, however, has a direct, Translin-independent mechanistic role as a scaffold protein in the DNA double-strand break (DSB) repair pathway by assisting the ATM kinase to establish an appropriate DNA damage response [23].

Here we use the RNAi-proficient model, *Schizosaccharomyces pombe*, to reveal a Trax- and RNAi-independent function for Translin (Tsn1) in maintaining genome stability in the absence of Dicer (Dcr1) and demonstrate that this is associated with a functional role in limiting retention of RNA polymerase II on the genomic template, which may serve to repress transcription-associated recombination. We extend this to show that this function is conserved in human Translin (TSN), revealing the first mechanistic link between Translin and disease-associated chromosomal recombination.

## Materials and methods

### Strains

A list of *S*. *pombe* strains used in this study can be found in S1 Table. Lineage of strains can be obtained from the corresponding author upon request. *S*. *pombe* cells were maintained and cultured in standard media [yeast extract liquid (YEL), yeast extract agar (YEA) or Edinburgh Minimal Medium + Glutamic Acid (20 mM) (EMMG)] as required with addition or omission of reagents as required/specified [24, 25]. Strain construction, storage, gene deletions and transformations used standard *S*. *pombe* protocols described by Bähler *et al*. [26], Forsburg and Rhind [24] and Sabatinos and Forsburg [25].

### Microscopic examination of mitosis

Single colonies of appropriate strains were inoculated into 5 ml YEL containing supplemental adenine (200 mg/ml) and incubated with rotation at 30°C until mid-log phase. Cells were harvested in a benchtop microfuge and resuspended in 1 ml 70% ethanol and incubated at room temperature for 10 minutes. Cells were re-harvested, and the pellets were washed three times with 1 ml phosphate-buffer saline (PBS; 137 mM NaCl, 2.7 mM KCl, 10 mM Na_2_HPO_4_, 1.8 mM KH_2_PO_4_). Cell were finally resuspended in 100 μl of PBS. 2 μl of cells were mixed with 2 μl DAPI (50 mg/ml) on a poly-L-lysine coated slide (Sigma, P8920) and air dried. Coverslip and a drop of Vectashield were applied prior to examination under a Zeiss Axioskop 2 plus fluorescence microscope. Slides were counted blind.

### Spot assays

Required strains were cultured in appropriate liquid media to mid log phase (OD_600_ of ~0.5). Cells were subjected to a 10-fold serial dilution (10^−1^–10^−5^) and 10 μl of each dilution were spotted onto agar plates of appropriate media. Plates were incubated for 3 days at 30°C unless otherwise stated.

### Estimation of mini chromosome instability

Ch^16-23R^ is a derivative of *S*. *pombe* Chromosome III and it carries the *ade6-M216* allele, which interallelically complements an *ade6-M210* allele located on the full-length Chromosome III [27–29]. Strains containing a mini chromosome *ade6-M216* allele and a full-length chromosome *ade6-M210* allele will be Ade+ (these cells produce colonies that are white on YEA plates without supplemental adenine, whereas cells that have lost the minichromosome and only carry the *ade6-M210* allele are Ade- and grow as red colonies); loss of the mini chromosome will result in Ade-. To calculate the rate of minichromosome loss we employed the method of Niwa [29]. In brief, appropriate strains containing the minichromosome were cultured in liquid EMMG medium containing appropriate supplements, but no adenine (for minichromosome maintenance selection). Cultures were incubated until late log-phase, subjected to serial dilutions, and then plated out onto YEA without supplemental adenine to give plates with approximately 100–200 colonies per plate. Loss of the minichromosome prior to plating will result in colonies that are completely red. Cells that retain the minichromsome in both daughter cells after the first mitotic division post-plating (i.e. one cell to two cell stage) will either be all white or have red/white sectored colonies with the ratio of white being greater than red (the latter arising due to minichromosome loss after the first division). Half sectored colonies (50% white and 50% red) represent a minichromosome loss event in the first division of the single colony forming cell. Counting the half-sectored colonies as a fraction of the total number of cells gives a relatively accurate approximation of the minichromosome loss rate per cell division (see Niwa [29] for further details and limitations). Colony colours were counted to quantify cells that had retained the minichromsome at the first post-plating division (white or sectored with the majority white), cells that did not contain the minichromosome at plating (totally red) and cells that had lost the minchromosome at the first division post-plating (half sectored 50:50 red:white). All strains were counted blind.

Colony counts were used to gain an approximate value for loss rate per cell division using the equation *N*_hs_/(*N*_total_*—N*_r_), where *N*_hs_ = number of half-sectored, *N*_total_ = total number of colonies, *N*_r_ = total number of all red colonies [29]. *P* values were calculated using Student’s *t*-test (two-tailed) and error bars represent standard deviation.

### Determination of recombination frequency

Appropriate *S*. *pombe* strains containing the plasmid pSRS5 [30] were cultured to mid-log phase in liquid EMMG medium containing appropriate supplements. Cells were subjected to serial dilution and plated out on EMMG agar containing appropriate supplements at a dilution that resulted in well-dispersed single colonies following incubation at 30°C. Colonies were permitted to grow until visible, but not permitted to reach greater than 1 mm in diameter. At this point a minimum of 7 whole colonies were individually picked and inoculated into individual 5 ml volumes of sterile liquid EMMG containing appropriate supplements, ensuring all the cells from the colony were transferred to the liquid medium. Cultures were incubated at 30°C with shaking until very early stationary phase. Serial dilutions were made for each culture and these were plated onto YEA (dilution range 10^−4^ to 10^−6^) and YEA containing 20 mg/ml guanine (pH 6.5) (dilution range neat– 10^−2^), which prevents the uptake of adenine because of purine antagonism [31]. Plates were incubated at 30°C for 3 days. All strains were counted blind.

The numbers of colony forming units per ml of culture were counted to determine recombination frequencies (Ade+ cells / 10^6^ viable cells). The recombination frequency was determined for 7 independent cultures for each strain to be tested and the median value was used for the recombination frequency (to avoid ‘jackpot’ values). This was repeated a minimum of three times for each strain to obtain mean values of independent biological repeats. P values were calculated using Student’s *t*-test (two-tailed) and error bars represent standard deviation.

### Gene overexpression

Genes were cloned into the *S*. *pombe* expression vector pREP3X [32] under the control of the regulatable *nmt* (no message in thiamine) promoter, which is repressed when cells are cultured in media containing thiamine [33]. pREP3X contains a *LEU2*^+^ marker gene for selection in *S*. *pombe* [32]. Strains containing genes cloned into pREP3X were cultured in liquid EMMG with appropriate strain-specific supplements and 2% thiamine, but without leucine (for plasmid maintenance). Cells were cultured in a rotary incubator at 30°C to mid log-phase (OD_600_ of ~0.5). For gene expression during spot analysis strains were spotted onto EMMG agar without thiamine. EMMG agar plates containing thiamine (2%) and YEA were used as a ‘gene off’ controls.

For *pac1*^+^ and *rnh1*^+^ overexpression analysis qRT–PCR was conducted using the QuantiTect SYBR Green PCR Kit (Qiagen; 204054) to amplify cDNA using a CFX96 real-time system (Bio-Rad) in accordance with the manufacturer’s instructions. For a 20 μl reaction mixture, the following were combined: 10 μl of SYBRTM Green master mix, 4 μl of the diluted cDNA (including 2.5 μl of nuclease-free water and 1.5 μl cDNA and), 4μl of 10 pmol/μl forward and reverse primers and 2 μl of sterile dH_2_O. Triplicate samples were prepared and loaded into PCR plates (BioRad) and the following PCR protocol was used: 3 minutes at 95°C, then 40 cycles at 95°C for 10 seconds, 30 seconds at 60°C, and 10 seconds at 95°C. *act1*^+^ was used for normalization.

### Cloning and site directed mutagenesis

RNA was extracted from *S*. *pombe* wild-type using the MasterPure Yeast RNA Purification Kit (Cambio; MPY03100). Human uterus RNA was obtained from Takara (Takara; 636551). cDNA was synthesized using the Superscript III First-Strand Synthesis System (ThermoFisher Scientific; 18080–051). Genes of interest were amplified from cDNA using primers shown in S2 Table. A *Bam*HI (New England Biolabs; R3136s) restriction site was added to each primer sequence for cloning into the pREP3X expression vector [32]. Amplification from human and *S*. *pombe* cDNA was done using Phusion High-Fidelity DNA polymerase (New England Biolabs; M0530S). DNA sequencing of both strands (performed by Eurofins Genomics) of the cloned genes of interest confirmed that no undesired mutations were introduced during the PCR and cloning procedures. Point mutations in human *TSN* and *S*. *pombe tsn1*^+^ were introduced using the QuickChange Lightning site-directed mutagenesis system (Agilent; 210515). Following the mutagenesis procedure all mutant genes were re-sequenced to ensure the correct mutations had been made and no additional mutations had been added.

### Genomic alkali lability assay

Appropriate *S*. *pombe* strains were cultured in YEL to mid log phase (~0.5 OD_600_). DNA was extracted using the Epicentre MasterPure Yeast DNA Purification Kit (Cambio; MPY80200). Either KOH or KCl was added to 1 μg of genomic DNA to a final concentration of 0.2 M in 40 μl volumes and incubated at 55°C for 2 hours. 6X loading buffer (alkaline; AlfaAesar; J62157) was added to the KOH-treated samples and 6X loading dye (non-alkaline; New England Biolabs; B7024s) was added to the KCl-treated samples. Alkali treated samples were loaded onto a 1% alkaline agarose gel (1% agarose, 1 mM EDTA, 50 mM NaOH) and run in alkaline electrophoresis buffer (1 mM EDTA, 50 mM NaOH). Electrophoresis of KCl treated samples was performed using a 1% agarose gel run in tris-borate-EDTA (TBE) buffer (130 mM tris [pH 7.6], 45 mM boric acid, 2.5 mM EDTA). Gels were run at 1 V/cm for 18 hours. Alkaline gels were neutralized by soaking in 1 M tris HCl (pH8.0) for 1 hour prior to staining with SYBR Gold (Thermo Fisher Scientific; S11494) and imaged on a UV transilluminator (BioRad; Molecular Imager Gel Doc XR System).

Quantification of undegraded genomic DNA intensity from alkali and native gels was performed using ImageQuant software. Values from the intensity of undegraded genomic DNA from alkali gels were normalized against the values of the chromosomal DNA in the native gel. *P* values were calculated using one sample Student’s *t*-test and error bars represent standard deviation.

### DNA:RNA immunoprecipitation (DRIP)

DNA extraction was performed as described in Forsburg and Rhind [24]. Genomic DNA was fragmented using *Dde*I (10 U/μg of DNA) for 2 hours at 37°C (New England Biolabs; R0175s). DNA samples were divided into two and one sample was treated with RNase H (New England Biolabs; M0297s) for 2 hours at 37°C, the other sample was left untreated. For DRIP, DNA samples were then incubated overnight at 4°C in immunoprecipitation (IP) buffer [100 mM MES (pH 6.6), 500 mM NaCl, 0.05% Triton, 2 mg/ml BSA] in the presence of Protein G-coupled Dynabeads (Life Technologies; 10003D) previously incubated with S9.6 antibody (Kerafast; ENH001) according to the manufacturer’s instructions. The beads were then washed three times in IP buffer. After two additional washes in Tris-EDTA (TE) buffer [10 mM tris-HCl (pH 8.0), 1 mM EDTA] the beads were resuspended in 10% Chelex resin (BioRad; 1421253) and incubated at 98°C for 5 minutes. The mixture was then incubated with 20 μg of proteinase K (Qiagen; 19131) at 43°C for 30 minutes and then at 98°C for 5 minutes. After centrifugation for 5 minutes at 6,000 r.p.m. in a benchtop microcentrifuge, DRIP-qPCR was performed using the supernatant.

Real-time PCR was performed using 25 ng of input DNA and 1/20 of the input immunoprecipitated DNA (above) in the presence of GoTaq Green Master Mix (Promega; A6002). Reactions were done in duplicate and standard curves were calculated on serial dilutions (100 ng– 0.1 ng) of input genomic DNA. IP enrichment was calculated relative to RNase H treated IP using the following formula: DRIP enrichment = {[IP amount (ng) (no RNase H) / input amount (ng) (no RNase H)] / [IP amount (ng) (+ RNase H) / input amount (ng) (+ RNase H)]}. The resulting values were then presented as a percentage of the wild-type value. Primer sequences are given in S2 Table.

Quantification for DRIP-qPCR was accomplished using Ct values and a standard curve of ten-fold dilutions of input genomic DNA from the wild-type strain. Experiments were performed in duplicates.

### Chromatin immunoprecipitation (ChIP)

Appropriate *S*. *pombe* strains were cultured in 50 ml of YEL to mid log-phase. Cells were crosslinked with 1% paraformaldehyde solution (Electron Microscopy Sciences; 15714–5) at room temperature for 15 minutes. Reactions were quenched by addition of 2 ml of 2.5 M glycine for 15 minutes at room temperature. Cells were harvested by centrifugation for 5 minutes at 1000 *g*, washed twice with ice-cold PBS and resuspended in 400 μl of Buffer A [50 mM HEPES (pH 7.5), 140 mM NaCl, 1 mM EDTA, 1% Triton X-100, 0.1% sodium deoxycholate] supplemented with 1 mM PMSF (Sigma; P78830-1G) and 1 x Halt protease inhibitors (ThermoFisher Scientific; 78430). After an addition of an equal volume of acid washed glass beads (Sigma; G8772-500G), cells were vortexed for 60 minutes at 4°C using a disruptor genie (Scientific Industries) with a Turbomix attachment. Lysates were recovered from the beads and sonicated using a bath Bioruptor Sonicator (Diagenode) at 30 seconds on followed by 30 seconds off for 10 minutes to obtain chromatin fragments in the range of 200–800 base pairs. The total volume was increased to 1 ml by addition of Buffer A and the sonicate was centrifuged at 4°C in a benchtop microfuge at 14,000 r.p.m. for 10 minutes. The soluble chromatin was retained.

20 μl of washed Dynabeads M-280 sheep anti-mouse IgG (Thermofisher Scientific; 11201D) were added to the chromatin sample and incubated for 2 hours at 4°C. 20 μl of the pre-cleared sample was kept for the ‘input’ fraction and the rest was incubated overnight at 4°C with 2 μg of anti-RNA polymerase II antibody (Abcam; ab5408) or in the absence of antibody. 20 μl of washed Dynabeads were added and after 2 hours at 4°C they were washed sequentially three times with Buffer A, twice with Buffer A with 500 mM NaCl, twice with 250 mM LiCl, 1% NP-40, 1% sodium deoxycholate, 1 mM EDTA, 10 mM tris-HCl (pH8.0) and twice with 10 mM Tris-HCl (pH 8.0), 1 mM EDTA (pH 8.0). The beads and ‘input’ were resuspended in 100 μl of 10% Chelex (BioRad; 1421253) and incubated at 98°C for 5 minutes. The mixture was then incubated with 20 μg of proteinase K (Qiagen; 19131) at 43°C for one hour and then at 98°C for 5 minutes. After centrifugation for 5 minutes at 6,000 r.p.m. in a benchtop microcentrifuge, the supernatant was collected and analyzed by qPCR (below).

qPCR was performed using the primers listed in S2 Table. Average CT was calculated across technical triplicates for each sample. Ct values for ChIP-qPCR were normalized using the Percent Input analysis method, which represents the amount of DNA pulled down by using the antibody of interest in the ChIP reaction, relative to the amount of starting material (‘input’ fraction). Experiments were done in triplicate. *P* values were calculated using Student’s unpaired *t*-test with Welch’s correction, and error bars represent standard deviation.

### Data package

The data used to generate plot figures within the manuscript and the supplemental materials is available at https://datadryad.org/stash/share/EZ6NdJ2zJdS8dCIepa60SaamaMcqS8Zv6F7UmrVpmyA

[34]

### Dryad DOI

https://datadryad.org/stash/share/EZ6NdJ2zJdS8dCIepa60SaamaMcqS8Zv6F7UmrVpmyA

## Results

### Tsn1 (Translin) is required to maintain genome stability in Dcr1 deficiency

**Fig 1:**
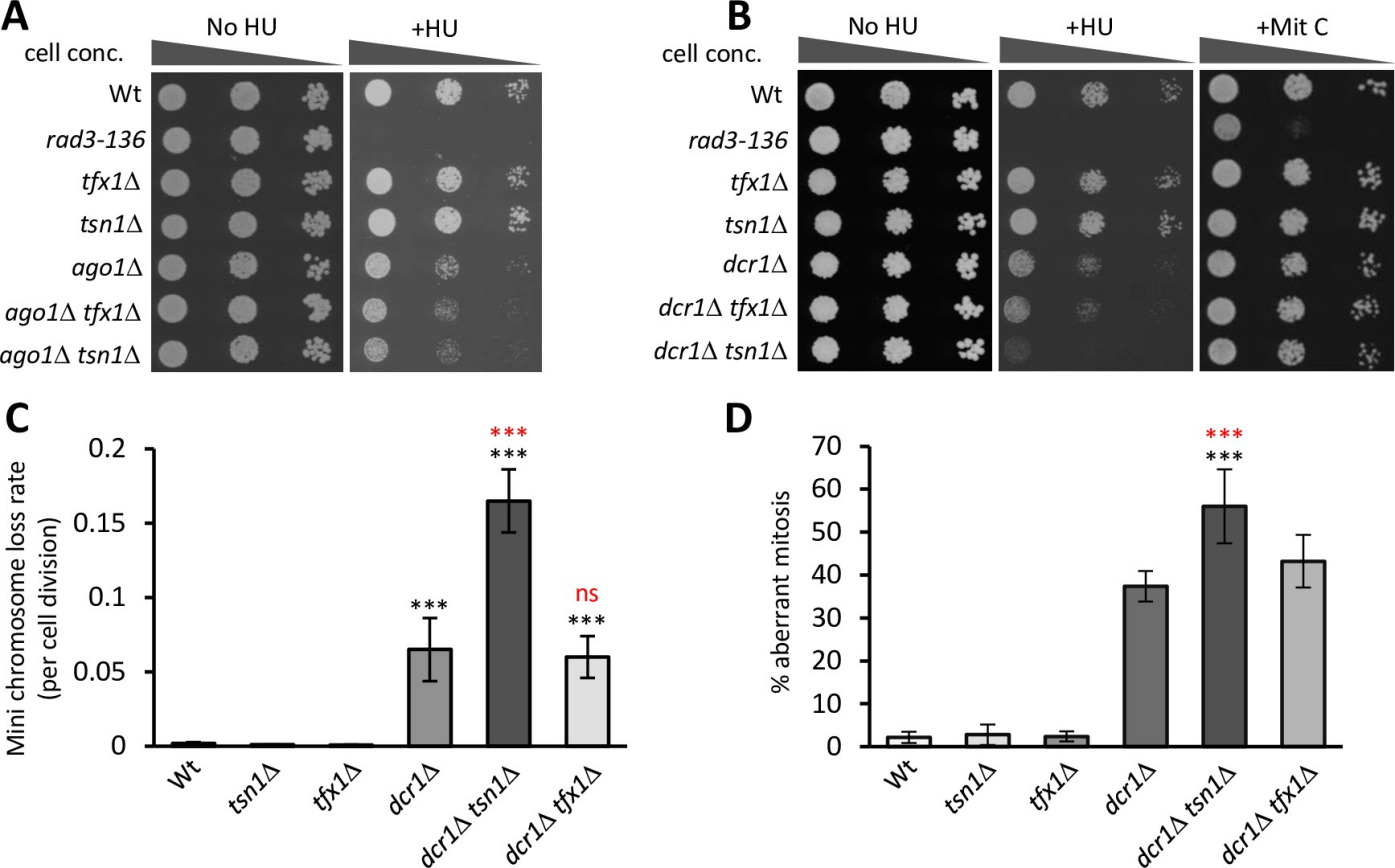
*tsn1*^+^, but not *tfx1*^+^, is required to maintain genome stability in Dcr1-deficiency, but not Ago1-deficiency. (A) Tsn1 and Tfx1 are not required for replicative stress response in Ago1-deficiency. 10-fold serial dilutions of indicated strains were spotted onto YEA with or without HU (10 mM). (B) Tsn1, but not Tfx1, function is required for some DNA replication stress responses, but not others, when Dcr1 function is lost. 10-fold serial dilutions of indicated strains were spotted onto YEA with or without HU (10 mM) or mitomycin C (150 nM). (C) Percentage of mini chromosome loss values show Tsn1 is required for chromosome stability in the absence of Dcr1. (D) Loss of Tsn1 function elevates the frequency of aberrant mitotic events in the absence of Dcr1. For C and D error bars represent standard deviation, ***P < 0.005 (black = Student’s *t*-test pairwise comparison with wild-type; red = Student’s *t*-test pairwise comparison with *dcr1*Δ mutant), ns = not significant; values are obtained from a minimum of five independent biological repeats.

Evidence for a direct role for Translin in genome stability maintenance is limited. *S*. *pombe* null mutants of either *tsn1*^*+*^ or *tfx1*^*+*^ exhibit no overt genome instability phenotype [35]. The link between Tn-Tx (C3PO) and RNAi in higher eukaryotes led us to explore whether there is a functional relationship between Tsn1 and/or Tfx1 and RNAi components Dcr1 and Ago1 in *S*. *pombe*. We recently noted that the sensitivity of the RNAi defective *ago1*Δ mutant to the microtubule destabilizing drug TBZ could be partially suppressed by mutating *tfx1*^*+*^, but not *tsn1*^*+*^, which was attributed to loss of a telomere-associated function of Tfx1 [36]. However, similar suppression is not observed for *ago1*Δ *tfx1*Δ double mutants exposed to the DNA replication inhibitor hydroxy urea (HU) (Fig 1A). Additionally, mutating *tsn1*^*+*^ or *tfx1*^*+*^ in the *ago1*Δ background does not increase HU sensitivity (Fig 1A), indicating there is no overt genetic interaction between *tsn1*^*+*^ or *tfx1*^*+*^ and the canonical RNAi pathway (which has an obligate requirement for Ago1) for replicative stress response.

The RNAi mediator Dcr1 has an RNAi-independent function (not Ago1-dependent) in genome stability maintenance [37–39]. Given this, and the oncogenic role for Tn-Tx in Dicer-deficient tumours [14], we tested *dcr1*Δ *tsn1*Δ and *dcr1*Δ *tfx1*Δ strains to assess potential genetic interaction between *dcr1*^*+*^ and either *tsn1*^*+*^ or *tfx1*^*+*^ for replicative stress response. Exposure of the *dcr1*Δ *tsn1*Δ double mutant to HU revealed that loss of *tsn1*^*+*^ in a *dcr1*Δ background increases sensitivity relative to the *dcr1*Δ mutant, indicative of a requirement for Tsn1 in Dcr1 deficiency (Fig 1B; increased sensitivity is suppressible by over expression of both *tsn1*^+^ and *dcr1*^+^, S1 Fig). There is no similar requirement for Tfx1 (Fig 1B), indicating this function of Tsn1 is not mediated by a Tn-Tx complex. We extended this by testing another DNA replication inhibitor, mitomycin C. No sensitivity was observed for any mutants (Fig 1B), indicating that Tsn1 and Dcr1 only regulate the response to specific replicative stresses.

Given that *S*. *pombe* Tsn1 does not appear to function in a Tn-Tx-like complex (consistent with previous findings [36]), we wanted to explore the functional overlap between the paralogous genes (*tsn1*^+^ and *tfx1*^+^) for genome stability response and so wild-type *tfx1*^*+*^ was over expressed in the *dcr1*Δ *tsn1*Δ double mutant. It did not suppress the increased HU sensitivity caused by the loss of *tsn1*^*+*^, confirming the functional independence of the paralogues (S2 Fig).

To determine whether Tsn1 functions to maintain genome stability in the absence of externally induced replicative stress and to offer a distinct measure of genome stability, we measured the stability of a mini, non-essential chromosome III derivative (Ch^16-23R^ [27,28]). *tsn1*Δ and *tfx1*Δ mutants both exhibit loss rates indistinguishable from the wild-type, but loss rates are elevated in the *dcr1*Δ mutant (Fig 1C). The *dcr1*Δ *tfx1*Δ strain loss rates are indistinguishable from the *dcr1*Δ mutant, whereas the *dcr1*Δ *tsn1*Δ double mutant exhibited a loss rate considerably higher than the *dcr1*Δ single mutant, indicating that without external insult Tsn1 is required to maintain genome stability in Dcr1 deficiency (Fig 1C).

*dcr1*Δ mutants also exhibit higher levels chromosomal structural anomalies during mitosis (asymmetric chromosome segregation / lagging chromosomal material) indicative of genome instability. Consistent with the mini chromosome instability data, we observed higher levels of mitotic chromosomal anomalies in the *dcr1*Δ *tsn1*Δ double mutant relative to the *dcr1*Δ single mutant. Again, consistent with the HU and mini chromosome stability assays the *dcr1*Δ *tfx1*Δ mutant exhibited levels similar to the *dcr1*Δ mutant (Fig 1D).

### tsn1^+^ *and* dcr1^+^ *exhibit epistasis with* rnh201^+^ *(RNase H2)*

Dcr1 acts independently of its RNase catalytic activity to protect genome stability by removing RNA polymerase II (RPII) from the DNA template, limiting transcription-replication conflicts (TRCs) [38]. In the absence of Dcr1 RPII retention has been reported to be associated with elevated RNA:DNA hybrids (R-loops) [38]. Unprocessed R-loops can present a challenge to genome stability by perturbing replicative progression [40–46]. However, R-loops can also serve a positive function in DSB repair, during which RPII and/or RNA Polymerase III (RPIII) synthesize transcripts, including so called damage-induced long non-coding RNAs (dilncRNAs), at DSB sites to actively contribute to the hierarchical phase regulated repair structures [47–49]. In all cases, R-loops must ultimately be removed to maintain genome integrity, and this is largely mediated by RNase H proteins [50]. In most eukaryotes there are two conserved and redundant RNase H activities, RNase H1 (Rnh1 in *S*. *pombe*) and RNase H2 (a heterotrimeric complex containing Rnh201 in *S*. *pombe*). In humans both RNase H activities are essential, although H2 is thought to be the predominant activity [51]. In *S*. *pombe* loss of both RNase H pathways results in loss of viability in response to replicative stress [52,53]. Whilst the two RNase H activities are largely redundant, the *S*. *pombe rnh201*Δ single mutant exhibits sensitivity to HU, but only under logarithmic growth conditions in rich media [53] (S3 Fig), possibly because Rnh201, but not Rnh1, is also required to remove mis-incorporated monoribonucleotides from DNA [50].

**Fig 2:**
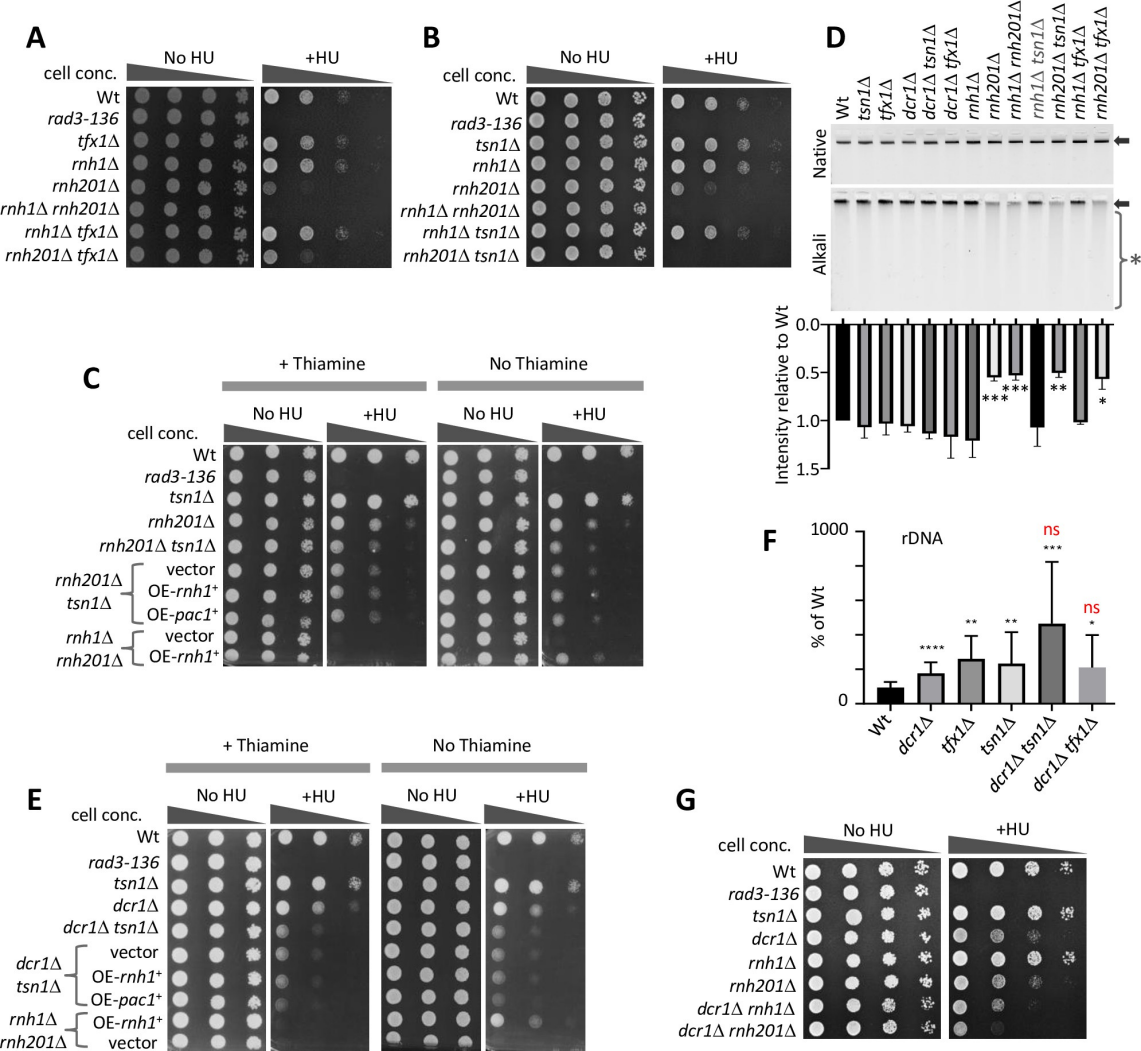
*tsn1*^+^ and *dcr1*^+^ are both functionally redundant with *rnh201*. (A) 10-fold serial dilutions of indicated strains were spotted onto YEA with or without HU (10 mM). (B) 10-fold serial dilutions of indicated strains were spotted onto YEA with or without HU (10 mM). (C) 10-fold serial dilutions of indicated strains were spotted onto EMM with thiamine (with and without HU; 10 mM) or without thiamine (with or without HU;10 mM). OE–overexpression (RT-qPCR showing overexpression is shown in S3 Fig). (D) Top: Alkali and native gels showing genomic DNA extracted from the indicated strains. Arrows indicate the genomic DNA band and the single asterisk indicates the region of degraded genomic DNA. Bottom: Quantification of genomic DNA band intensity from the alkali gel (values normalized against the intensity of the chromosomal DNA band in the native gel). *P < 0.05, **P < 0.01, ***P < 0.001 from Student’s *t*-test in pairwise comparisons relative to wild-type. Bars represent standard deviation. Values are obtained from a minimum of three independent biological repeats. (E) 10-fold serial dilutions of indicated strains were spotted onto EMM with thiamine (with and without HU; 10 mM) or without thiamine (with or without HU;10 mM). OE–overexpression (RT-qPCR showing overexpression is shown in S3 Fig). (F) Quantification of DRIP for the rDNA (18S) locus for the indicated strains. *P < 0.05, **P < 0.01, ***P < 0.001, ns–not significant, from Student’s *t*-test in pairwise comparisons relative to relative to wild-type (black) or to *dcr1Δ* (red). Bars represent standard deviation. Values are obtained from a minimum of three independent biological repeats. (G) 10-fold serial dilutions of indicated strains were spotted onto YEA with or without HU (10 mM). Note: distinct relative sensitivities are observed when mutants are grown on minimal media (EMM) *vs*. rich media (YEA), which is due to distinct growth rates. OE-Sp*tsn+*—overexpressed *S*. *pombe tsn1*^+^; OE-Sp*tsn1-E152A –*overexpression of *S*. *pombe tsn1*^+^ mutated at sole the residue conserved with the Tfx1 (and TSNAX) RNase catalytic domain.

Given that the loss of Dcr1 causes R-loop accumulation, we next explored the relationship between Tsn1, Tfx1 and Dcr1 and the two RNase H pathways. We firstly constructed double mutants of both *tsn1*^*+*^ and *tfx1*^*+*^ with null mutants of both core RNase H (1 and 2) encoding genes, *rnh1*^*+*^ and *rnh201*^*+*^. The *rnh1*Δ *tfx1*Δ and *rnh201*Δ *tfx1*Δ double mutants exhibited no increased sensitivity to HU (Fig 2A). However, the *rnh201*Δ *tsn1*Δ double mutant, but not the *rnh1*Δ *tsn1*Δ double mutant, exhibited considerable HU sensitivity (Fig 2B), indicating that *tsn1*^+^ is epistatic with *rnh201*^+^.

RNase H2 can process R-loops and *Saccharomyces cerevisiae* RNase H1 and H2 appear to have distinct roles in R-loop processing [54,55], whilst in humans RNase H2 provides the predominant activity [51]. However, unlike RNase H1, RNase H2 also has the ability to remove mis-incorporated ribonucleotides [50]. Based on our data (above) and the known roles for RNase H2, we hypothesized two possibilities for Tsn1. Firstly, it facilitates RNase H1-mediated R-loop removal (via an RNase activity). Secondly, it could function redundantly with Rnh201 in mis-incorporated ribonucleotide removal. To test the first possibility, we overexpressed the RNase H1 gene, *rnh1*^*+*^, under a thiamine repressible promoter (*nmt*) plasmid in the *rnh201*Δ *tsn1*Δ double mutant, to assess whether the inability to cope with the replicative stress could be suppressed by elevated levels of R-loop processing RNase H activity. Over expression of *rnh1*^*+*^ (no thiamine) did not alleviate the inability of the *rnh201*Δ *tsn1*Δ double mutant to tolerate HU but did suppress the HU sensitivity of the *rnh1*Δ *rnh201*Δ double mutant back to *rnh201*Δ single mutant levels, which indicates functional Rnh1 production from the plasmid (Figs 2C and S4A).

The Tn-Tx complex (C3PO) utilizes ribonuclease activity for RNAi passenger strand processing, with the catalytic activity being mediated by Trax and not Translin [7,8]. So, despite an uncorroborated report of exceptionally weak ribonuclease activity for recombinant mammalian Translin [56], it is likely that Tsn1 (independent of Tfx1) functions to mediate genome stability maintenance in an RNase-independent fashion, as is the case for Dcr1 [38]. This is further supported by the fact that the amino acids required for RNase catalytic activity of Trax are mostly poorly conserved in Translin, with the only one human Trax RNase catalytic residue being conserved between Trax and Translin [human Trax Glu197; also conserved in *S*. *pombe* Tns1 (Glu152); S5A Fig] [7,8,57,58], which is not required for Tsn1 function [we generated a *tsn1-E152A* mutant, over expression of which rescued the *tsn1*Δ phenotype to a level equivalent to *tsn1*^+^ over expression (S5B Fig)]. However, mammalian Dicer and Translin are associated with RNase activities on RNA duplex substrates, so to ensure that the R-loops do not have a dsRNA component which require degradation for R-loop processing, we also explore whether over expression of a prominent dsRNA specific RNase gene, *pac1*^+^, could suppress the requirement for Tsn1. It did not (Figs 2C and S4B). Together these data strongly suggest that Tsn1 is not functioning via a dsRNase/RNase H activity.

To test the second possibility, that Tsn1 might function in a secondary pathway for mis-incorporated ribonucleotide removal, we assessed ribonucleotide levels in genomic DNA in distinct mutants using alkali gel electrophoresis; ribonucleotides in DNA are labile under alkali gel conditions, resulting in quantifiable loss of chromosomal DNA intensity on an alkali gel verses a neutral gel. Chromosomal DNA from null mutants of *rnh201*^*+*^ exhibited elevated alkali-dependent degradation (Fig 2D). However, mutation of *tsn1*^*+*^, alone or in a *rnh201*Δ background, did not increase alkali-dependent sensitivity, indicating there is no discernable increase in chromosomal ribonucleotides in response to *tsn1*^*+*^ loss (Fig 2D).

In *S*. *cerevisiae* over production of Rnh201 alone can suppress defects caused by the loss of Rat1, a 5′ to 3′ RNA exonuclease, indicting Rnh201 can suppress other RNA processing activities [59]. To determine if Tsn1 functions redundantly for processing another undetermined Rnh201 substrate, we over expressed *rnh201*^+^ in the *dcr1Δ tsn1Δ* mutant to assess whether Rnh201 over production could suppress HU sensitivity. This was not the case (S4C Fig), indicating that there is no biochemical mechanistic overlap between Rnh201 and Tsn1. Together, these findings indicate that Tsn1 does not function either as a ribonuclease (dsRNAs or RNase H), nor in the excision of mis-incorporated ribonucleotides.

That the *rnh201*Δ *tsn1*Δ strain HU sensitivity could not be suppressed by over expression of *rnh1*^+^ (RNase H1) indicates replicative stress intolerance due to loss of Tsn1 is not due to elevated R-loops that cannot be tolerated. However, *dcr1*Δ mutants do accumulate R-loops [38]. To further explore the possibility that the role of Tsn1 in the *dcr1*Δ background could be to remove R-loops, we also over expressed *rnh1*^*+*^ in the *dcr1*Δ *tsn1Δ* strain. This also failed to rescue the HU sensitivity (Figs 2E and S4A), indicating that unprocessed R-loops are not causing the replicative stress sensitivity. As Dcr1 has dsRNA specific RNase activity, we also over expressed the *pac1*^*+*^ ribonuclease gene in the *dcr1*Δ *tsn1*Δ double mutant, but this too failed to suppress the HU sensitivity (Figs 2E and S4B). Interestingly, over expression of *rnh1*^*+*^ (or *pac1*^+^) also failed to rescue the HU sensitivity of the *dcr1*Δ single mutant in which R-loops accumulate (S4 Fig). Together these findings indicate replicative stress sensitivity is not caused by accumulation of R-loops or dsRNA, consistent with proposal that Dcr1 functions to maintain genome stability by an RNase-independent RPII template displacement mechanism [38].

Extending this, we used DNA:RNA-immunoprecipitation (DRIP) to directly measure R-loops at the rDNA locus and a tDNA gene, both of which have been reported to accumulate R-loops in *dcr1*Δ cells [38] (although the quantification of levels of R-loops reported for these loci was likely confounded to some degree by interference in the DRIP caused by the S9.6 antibody binding to dsRNAs [60]). As expected, we observed increased R-loops at both loci in the *dcr1*Δ mutant (Figs 2F and S6). However, surprisingly, R-loop levels were increased to the levels observed in the *dcr1*Δ mutant in both the *tsn1*Δ and *tfx1*Δ single mutants (Figs 2F and S6), in the absence of elevated transcripts from these loci [36], indicating both genes are required to minimize R-loop accumulation, at least at these loci. The *dcr1*Δ *tsn1*Δ and *dcr1*Δ *tfx1*Δ double mutants did not show a significant increase in R-loops relative to the respective single mutants (Figs 2F and S6). These data appear to indicate that there is no correlation between R-loop levels and sensitivity to replicative stress (caused by HU). However, the *dcr1*Δ *tsn1*Δ double mutant does exhibit an elevated, non-significant mean relative to *dcr1*Δ cells, albeit with a wide range, which could reflect a possible underlying epigenetic variability in this background. Moreover, previous extensive analysis of *tsn1*Δ and *tfx1*Δ single mutants has not revealed any indication of genome instability [35]. So, R-loops at these levels alone are not sufficient to cause deleterious genome instability or a failure to cope with replicative stress in these mutants, supporting the data obtained from over expressing *rnh1*^*+*^.

The genetic data suggest that *tsn1*^*+*^ and *rnh201*^*+*^ operate in distinct pathways, as is the case for *tsn1*^*+*^ and *dcr1*^*+*^. Together with the fact that Rnh1 and Rnh201 function redundantly for R-loop processing, one interpretation of our findings is that Tsn1 functions in a pathway with Rnh1 (although *rnh1*^*+*^ overexpression does not support this being RNase H mediated), and so this led us to postulate that Dcr1 functions in a Rnh201-depedent pathway (i.e. Tsn1/Rnh1 function in one pathway and Dcr1/Rnh201 function in another, both pathways being redundant). This hypothesis predicts that the *dcr1*Δ *rnh1*Δ strain will be hypersensitive to HU and the *dcr1*Δ *rnh201*Δ strain will not be. We constructed the appropriate mutants and found that this hypothesis does not hold. Indeed, the *dcr1*Δ *rnh201*Δ double mutant was exquisitely sensitive to HU and the *dcr1*Δ *rnh1*Δ exhibited HU sensitivity similar to the *dcr1*Δ single mutant (Fig 2G). Together, these data indicate that Tsn1 and Dcr1 could function redundantly with Rnh201 and with one another. We believe it is unlikely that these additive effects are due to summative defects in unrelated pathways as the *tsn1*Δ single mutant has no overt genome instability defects, although we cannot rule this possibility out altogether.

### Tsn1 functions to tolerate replicative stress independently of telomeres

**Fig 3:**
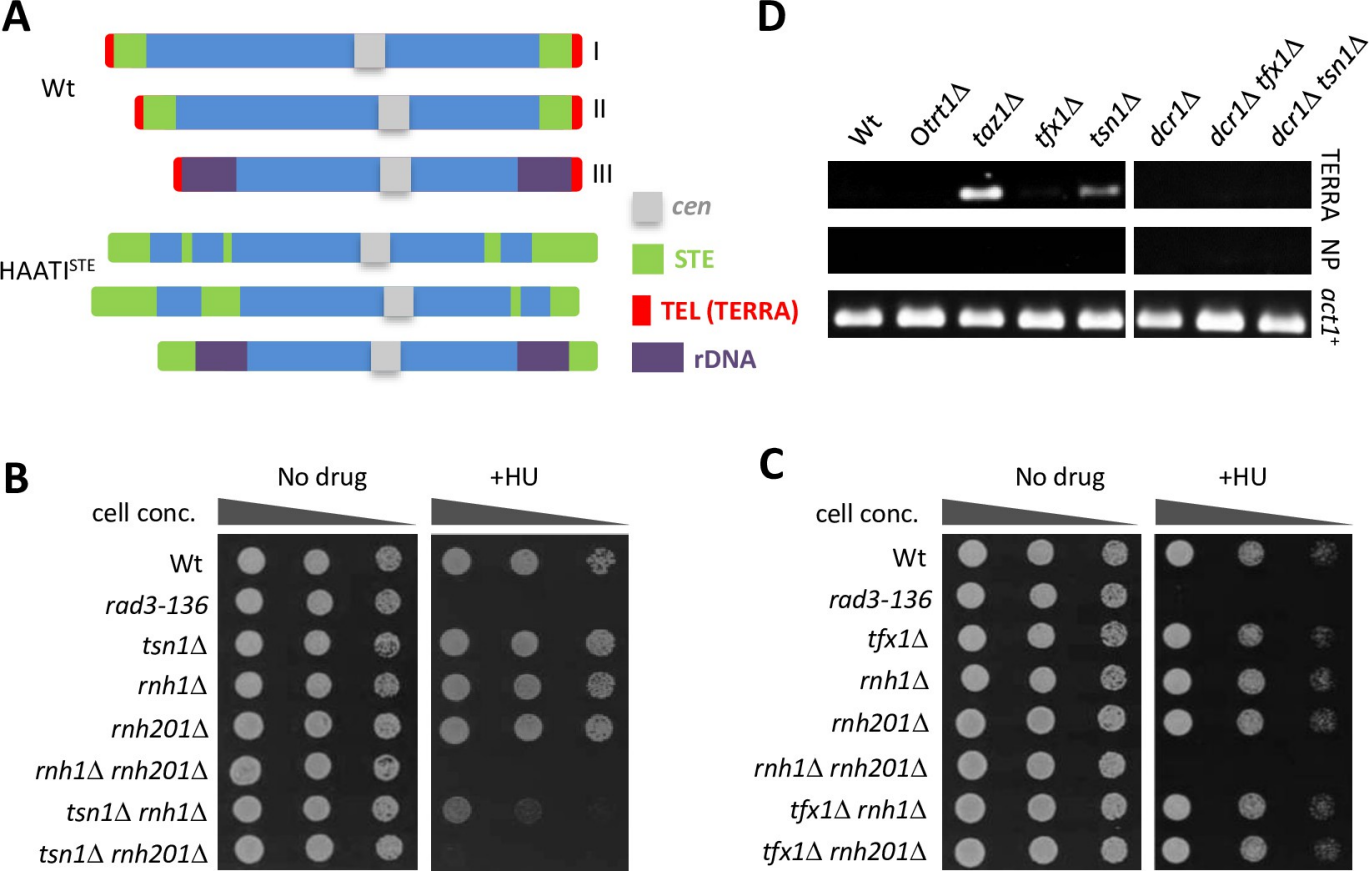
*tsn1*^+^, but *not tfx1*^+^, is required for replicative stress response in Dcr1-deficiency in the absence of telomeres. (A) Schematic of the *S*. *pombe* chromosome structure for wild-type (Wt) and HAATI^STE^ strain. The three Wt chromosomes are shown on the top (I, II and III represent the Wt chromosome designations); the three HAATI^STE^ chromosomes are shown on the bottom [61]. *cen* = centromere; STE = subtelomeric elements; TEL = telomere, template for TERRAs; rDNA = ribosomal DNA. (B) 10-fold serial dilutions of indicated strains were spotted onto YEA with or without HU (10 mM). (C) 10-fold serial dilutions of indicated strains were spotted onto YEA with or without HU (10 mM). (D) Agarose gel images showing reverse transcriptase PCR products for RNA extracted from the indicated strains. Left hand set is as previously reported [35]; right hand set from this study. *taz1*Δ–control for elevated TERRA; O*trt1*Δ–no telomere control. NP–No primer control. Primers to *act1*^+^ used as cDNA positive control.

Loss of Tsn1 function results in elevated levels of telomeric transcripts termed TERRAs, which are associated with DNA damage tolerance [36]. It is plausible that the requirement for Tsn1 in replicative stress tolerance is via TERRA regulation. To test this, we examined viable strains of *S*. *pombe* that lack telomeres, so called HAATI^STE^ cells, that have linear telomere sequences replaced with stretches of subtelomeric elements (STEs; Fig 3A; [61]) and will not have canonical TERRAs [62]. To check the need for Tsn1 in the telomere-free background we constructed *rnh1*Δ *tsn1*Δ and *rnh201*Δ *tsn1*Δ HAATI^STE^ mutants (we did not use the *dcr1Δ* background as Dcr1 has a role in regulation of sub-telomeric regions). Similar to telomere-proficient cells, the HAATI^STE^ *rnh201*Δ *tsn1*Δ double mutant exhibited considerable sensitivity to HU, comparable to the *rnh1*Δ *rnh201*Δ strain (Fig 3B). This demonstrates a requirement for Tsn1 in the absence of telomeres and canonical TERRAs. As for the telomere proficient cells, HAATI^STE^ strains exhibited no requirement for Tfx1 (Fig 3C).

Interestingly, the *rnh1*Δ *tsn1*Δ double mutant also exhibited a mild sensitivity to HU in this background, which is not observed in the telomere-proficient cells, but this was not as marked as the *rnh201*Δ *tsn1*Δ double mutant. The meaning of this observations is currently unclear, but it suggests that without telomeres there are distinct requirements for the two RNase H pathways. Also, the *rnh201*Δ single mutant does not appear to exhibit the HU sensitivity observed in the telomere proficient strains, which we believe is due to the distinct growth rate of the HAATI strains, as such distinct HU sensitivities have been previously reported for *rnh201*Δ mutants from different groups [52,53].

Further support for the role of Tsn1 in Dcr1 deficiency not being driven by elevated TERRAs comes from the unexpected finding that in telomere proficient cells mutation of *dcr1*^+^ suppresses the elevated TERRA levels observed in the *tsn1*Δ mutant (Fig 3D). Whilst the biological relevance of this distinct Dcr1-Tsn1 relationship at telomeres remains unclear, it offers additional support to the idea that the role of Tsn1 in genome stability maintenance is independent of excessive TERRAs.

### Tsn1 is required to suppress transcription-associated inter-molecular recombination

The RNAi-independent function of Dcr1 mediates the removal of RPII from genomic regions, including tDNAs [38]; the functional role of RPII at tDNAs is unknown, but R-loops, which could be generated by RPIII at tDNAs, serve as intrinsic RPII promoters [63]. tDNAs can slow replicative progression and tDNA rich sites are overrepresented at sites of genomic rearrangements, including translocations [64,65]. These factors led us to hypothesize that in the absence of Dcr1, Tsn1 is required to suppress inter-molecular recombination at loci which required Dcr1 to eject RPII, such as tDNAs.

**Fig 4:**
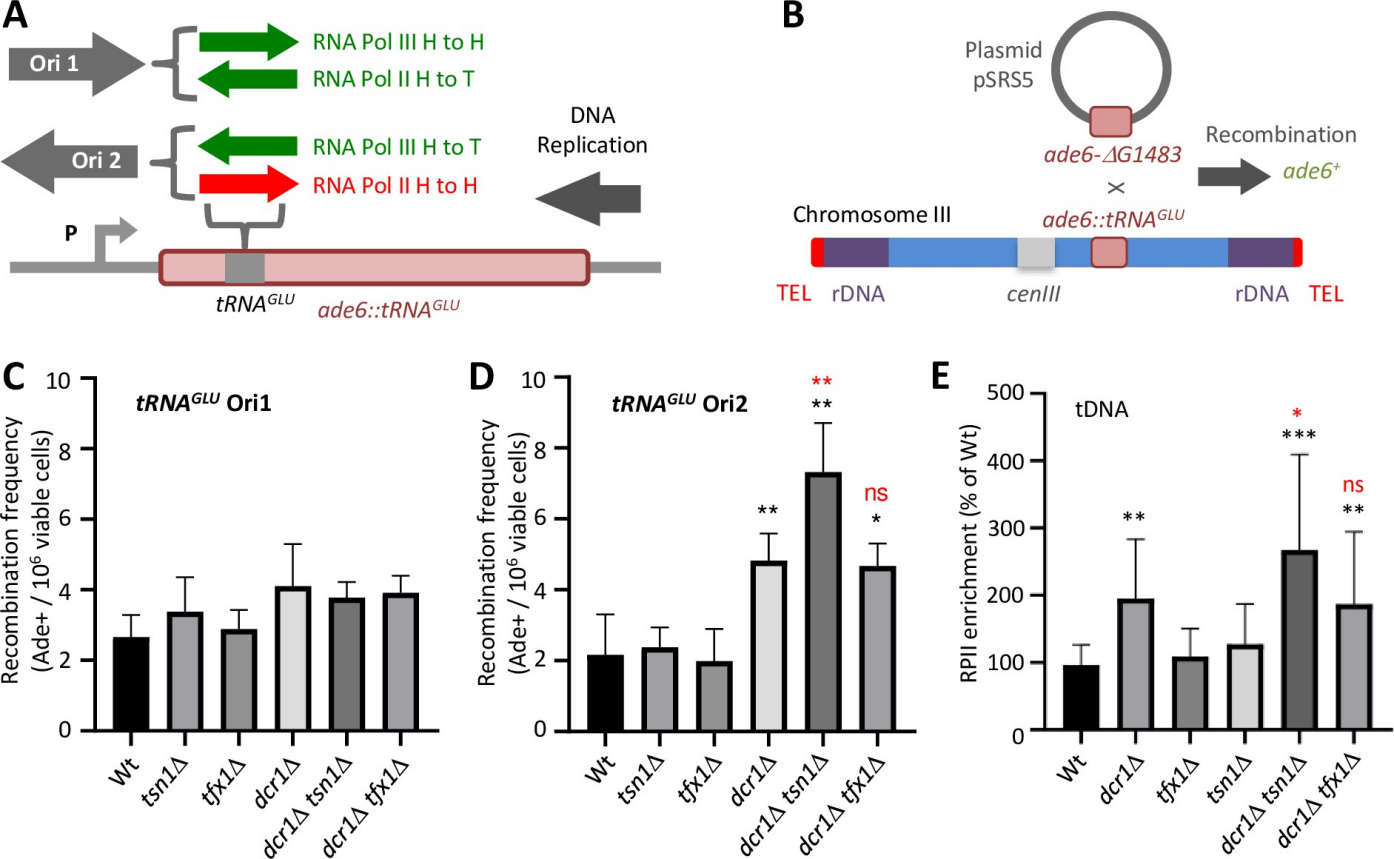
*tsn1*^+^ is required to suppress recombination in a polar fashion at a tDNA and to displace RPII in Dcr1-deficiency. (A) Schematic of the *ade6*::*tRNA*^*GLU*^ allele showing the approximate position of the tDNAs inserted into the *ade6*^+^ gene in different configurations (Ori1 and Ori2) (30). The orientation that gives an RPII head-to-head TRC is shown in red. (B) Schematic of the intermolecular recombination assay. (C) Quantification of recombination frequency for *tRNA*^*GLU*^ Ori1 for the indicated strains. Bars represent standard deviation. Values are obtained from a minimum of three independent biological repeats. (D) Quantification of recombination frequency for *tRNA*^*GLU*^ Ori2 for the indicated strains. *P < 0.05, **P < 0.01, ns–not significant from *t*-test in pairwise comparisons relative to relative to the wild-type (black) or to *dcr1*Δ (red). Bars represent standard deviation. Values are obtained from a minimum of three independent biological repeats. (E) Quantification of a tDNA gene (*tDNA*^*HIS*^) RPII ChIP for the indicated strains. *P < 0.05, **P < 0.01, ***P < 0.001, ns–not significant, relative to the wild-type (black) or to *dcr1*Δ (red). Bars represent standard deviation. Values are obtained from a minimum of three independent biological repeats.

To test this, we took advantage of the fact that tDNAs can be recombinogenic in *S*. *pombe* when recombination and replication systems are defective [30,66,67]. We employed an established inter-molecular recombination assay, which involves a tDNA inserted into the genomic *ade6*^*+*^ gene. In this case, *tRNA*^*GLU*^ is inserted into one of two orientations in distinct constructs (*tRNA*^*GLU*^ Ori1 and *tRNA*^*GLU*^ Ori2; Fig 4A) [30]. The *ade6*^*+*^ locus is predominantly replicated unidirectionally (Fig 4A) and the *ade6*::*tRNA*^*GLU*^ (Ori1 and Ori2) constructs serve as replicative pause sites, with neither orientation having greater pausing capacity than the other [30]. In the presence of an additional plasmid-borne mutant allele of *ade6*^*+*^, *ade6-ΔG1483*, gene conversion via inter-molecular recombination can generate *ade6*^*+*^ cells, enabling gene conversion frequency to be measured via fluctuation analysis (*ade6*^*+*^ cells can be detected by plating cells on guanine containing media [31]) (Fig 4B) (this assay does not distinguish between gene conversion events with or without crossover events and aims only to measure inter-molecular recombination events by scoring gene conversions not confounded by intra-chromatid / inter-sister chromatid events, which is the case for some recombination assays).

For *tRNA*^*GLU*^ in the two orientations RPII and RPIII will transcribe in opposing orientations, presenting distinct head-to-head RNA polymerase challenges to the replisome. For Ori1, RPIII will be in a head-to-head configuration with the DNA replisome and in Ori 2 RPII will be in the head-to-head configuration (Fig 4A).

Mutants were constructed to assess inter-molecular recombination frequencies in the absence of *dcr1*^*+*^, *tsn1*^*+*^ and *tfx1*^*+*^ for both orientations of *tRNA*^*GLU*^. For Ori1 (RPIII in head-to-head conflict with the replisome) none of the mutants exhibited difference in recombination frequency relative to the wild-type (Fig 4C). However, for Ori2 (RPII in head-to-head conflict with the replisome) loss of *dcr1*^*+*^ results in a significant elevation in recombination frequency greater than 2-fold relative to the wild-type, consistent with the prediction that Dcr1 is required for RPII displacement to prevent TRCs (Fig 4D; [38]). The *tsn1*Δ and *tfx1*Δ single mutants exhibit no elevation relative to the wild-type (despite elevated R-loops). Whilst the *dcr1*Δ *tfx1*Δ double mutant exhibited levels similar to the *dcr1*Δ single mutant, the *dcr1*Δ *tsn1*Δ double mutant exhibited a statistically significant elevation relative to the *dcr1*Δ single mutant (Fig 4D). These data are consistent with the pattern of HU sensitivity and the mini chromosome loss data, demonstrating that loss of Tsn1 in Dcr1 deficiency elevates recombination associated with a head-to-head RPII TRC, but not a RPIII TRC. This would indicate that replicative pauses *per se* do not require Tsn1 and/or Dcr1 function to suppress recombination, but those associated with RPII head-to-head TRCs do. However, it is important to note that for Ori2 there is also the potential for RPII transcribing from the endogenous *ade6*^+^ promoter (P in Fig 4A) to collide head-to-head with the *tRNA*^*GLU*^ RPIII, so we cannot exclude the possibility that the recombinogenic lesion is generated by a RPII-RPIII transcription-transcription conflict (TTC), or a combination of TTC/TRC. Whatever the case, the orientation-dependent nature of the recombination increase appears to demonstrate that this is a transcription-associated phenomenon.

### Tsn1 is required for RPII template displacement

The epistasis between *dcr1*^+^ and *tsn1*^+^ and the polar nature of recombination at a tDNA could suggest that Tsn1 functions in an auxiliary mechanism for template dissociation of RPII to prevent TRCs, the consequences of which become exacerbated upon external replicative stress (e.g., HU). To test this, we assessed RPII occupancy using chromatin immunoprecipitation (ChIP) of RPII at loci previously demonstrated to require Dcr1 for RPII displacement, rDNA and tDNA loci [38]. At both genomic elements, loss of *dcr1*^+^ resulted in elevated RPII retention, consistent with the findings of Castel and co-workers [38]. Loss of *tsn1*^+^ and *tfx1*^+^ did not increase RPII occupancy (Figs 4E and S7), despite loss of these two genes causing elevated R-loops (Fig 2F). However, when Tsn1 function is lost in the *dcr1*Δ background there is a significant rise in the levels of RPII retained on the templated relative to the *dcr1*Δ mutant for both genomic regions (Figs 4E and S7). This elevation is not observed when *tfx1*Δ is mutated in the *dcr1*Δ background (Figs 4E and S7), indicating that Tsn1, but not Tfx1, functions to mediate an auxiliary RPII displacement mechanism in Dcr1 deficiency.

### Human TSN functions to mediate genomic stress tolerance

**Fig 5:**
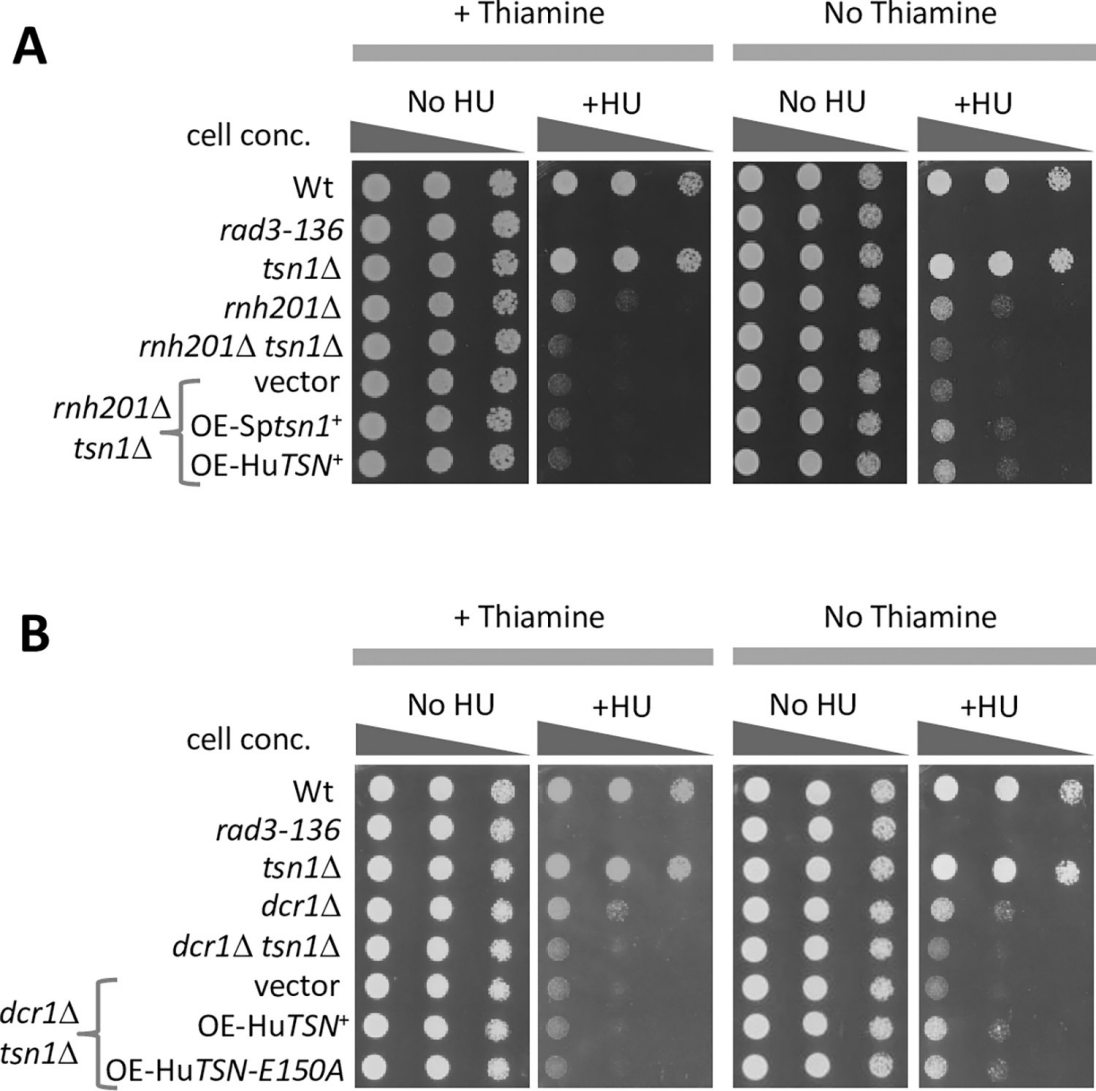
Human TSN functions to suppress the requirement for *S*. *pombe tsn1*^+^ in genome stability maintenance. (A) 10-fold serial dilutions of indicated strains were spotted onto EMM with thiamine (with and without HU; 10 mM) or without thiamine (with or without HU;10 mM). OE-Sp*tsn+*—overexpressed *S*. *pombe tsn1*^+^; OE-Hu*TSN*^*+*^—overexpressed human *TSN*^+^. (B) 10-fold serial dilutions of indicated strains were spotted onto EMM with thiamine (with and without HU; 10 mM) or without thiamine (with or without HU;10 mM). OE-Hu*TSN*^*+*^—overexpressed human *TSN*^+^; OE-Hu*TSN-E150A –*overexpression of human *TSN*^+^ mutated at sole the residue conserved with the TSNAX (and Tfx1) RNase catalytic domain.

Evolutionary conservation of Translin orthologue functions is evidenced by the fact that Tsn1 is required to maintain the stability of Tfx1 in *S*. *pombe* and mammals [35,68]. However, there is no evidence for a Tn-Tx-like complex (C3PO) in *S*. *pombe* and Tsn1 and Tfx1 do not appear to be needed for centromeric heterochromatin formation, suggesting that they are not essential for canonical RNAi [36]. This brings into question the relevance of this system for understanding human Translin (TSN) function. To directly address whether human TSN can function to maintain genome stability in response to genomic stress, we cloned the human *TSN* gene and over expressed it in the *dcr1*Δ *tsn1*Δ and *rnh201*Δ *tsn1*Δ double mutants. The expression of *TSN* suppressed the HU sensitivity of the double mutants to the same extent observed for *S*. *pombe tsn1*^*+*^ (Figs 5 and S1) [the same is observed for a *TSN* mutated in the amino acid conserved with the one of the TSNAX RNase catalytic amino acid residues (*TSN-E150A*), confirming this residue is not functionally important; Fig 5B]. Over expression of human *TSNAX* (Trax coding gene) did not suppress the HU sensitivity of the *dcr1*Δ *tsn1*Δ double mutant (S2 Fig), so human TSN could contribute to maintaining genome stability in a TSNAX-independent fashion, indicating functional independence of Translin for genome stability maintenance is potentially apparent in humans.

## Discussion

Since its discovery in humans as a chromosomal breakpoint junction binding protein [4] and in mice as Testis-Brain RNA binding protein [69,70], Translin has been implicated in a diverse range of fundamental biological processes, ranging from neurological regulation, including sleep and behavioural control (for examples, see [71–73]), through to oncogenic activity [14] and control of adiposity [74]. In many cases, Translin operates in a heterocomplex with Trax, but insight into independent functions is emerging. Moreover, Archaea only have one Translin/Trax paralogue, which is thought to be more similar to Trax, indicating the earliest function(s) to evolve were provided by one protein [58]. The data presented here support previous findings that *S*. *pombe* Tsn1 and Tfx1 do not function together as a complex, despite a functional association in telomere transcript control [36], suggesting that C3PO-like RNAi activity evolved later. However, here we demonstrate that a Trax-independent Translin function prevents genome instability in the absence of Dicer and this has been evolutionarily conserved from lower eukaryotes to humans, unlike the RNAi function of Translin. It is noteworthy, however, that it is known that the stability of Trax is dependent upon Translin in mammalian cells, a feature conserved in *S*. *pombe*, where there is a substantial reduction in Trax levels upon loss of Translin [35]. Given this, we cannot dismiss the possibility that this loss of Tfx1 in the *tsn1*Δ mutant contributes to the *tsn1*Δ observed phenotypes (despite the *tfx1*Δ mutant having no overt phenotype). For example, it remains a formal possibility that wild-type levels of Tfx1 (which are not present in the *tsn1*Δ mutant) could compensate for the loss of Tsn1 (but the *tfx1*Δ mutant shows no phenotype because function is primarily provided by Tsn1). If this were the case it might be expected that that *tfx1*^+^ overexpression would suppress the phenotype, which it does not (S2 Fig); however, if overexpression does not restore the exact wild-type Tfx1 levels or configuration (e.g., a modified version of Tfx1), then this could account for the lack of suppression of the *tsn1*Δ defect. Whilst this seems unlikely, the paralogous nature of these two proteins means this cannot be dismissed.

Dicer deficiency is oncogenic in many cancers and is linked to poor prognosis [75]; moreover, a number of cancers carry mutations in *DICER1* (for example, see [76]). A primary tumour suppressing role of Dicer is to process miRNA precursors in the cytoplasm. However, evidence is emerging to indicate that Dicer has nuclear function in response to DNA damage and replicative stress, which includes the processing of damage induced RNAs required for hierarchical DSB repair factor recruitment [77–79]. These findings, added to the fact Dicer has RPII dissociation function, indicates the oncogenic nature of limited Dicer may extend beyond the loss of pre-miRNA maturation. Translin, in complex with Trax, shares pre-miRNA substrate capabilities with Dicer, but this is only oncogenic when Dicer is compromised [14]. Here we now show that when Dicer is compromised, Translin, but not Trax, has additional functions, which could influence oncogenesis.

The mechanism by which Dcr1 removes RPII remains unclear, although it is independent of its RNase activity. Human Translin can compete for dilncRNAs with Dicer, but this function is likely to require the C3PO (Tn-Tx complex) RNase activity [47], so it is doubtful that this is directly linked to RPII displacement as this function is RNase-independent. The function of Tsn1 is also unlikely to be indirect via regulation of other transcripts, as *tsn1*Δ mutants exhibit no transcript level changes relative to the wild-type, other than TERRAs [36], which we demonstrate are not essential for the replicative stress tolerance function of Tsn1.

**Fig 6:**
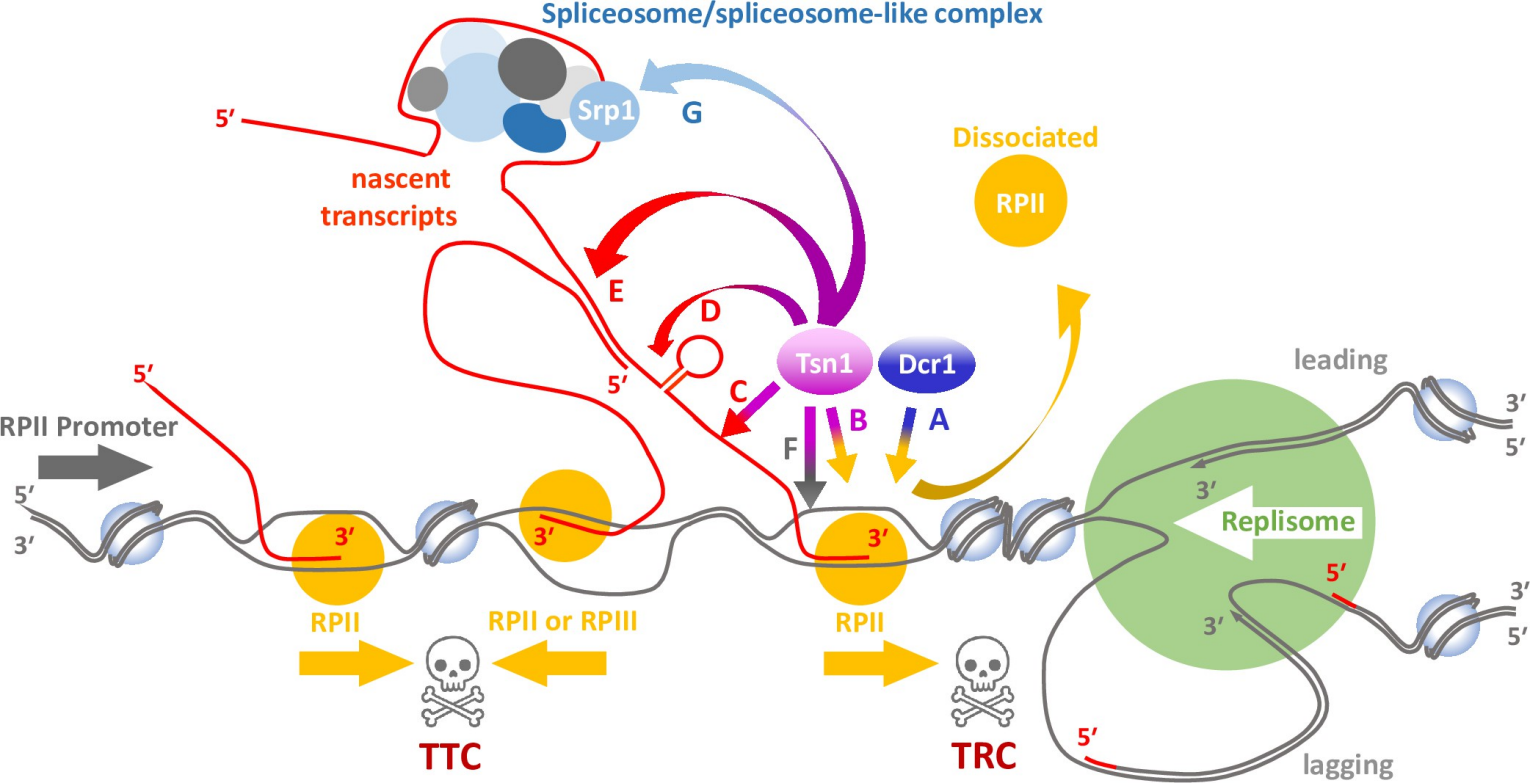
Possible mechanisms for the contribution of Tsn1 for RPII template displacement. The schematic represents potential evens at the *ade6*::*tRNA* site shown in Fig 4A to illustrate potential Tsn1 mechanisms of action. It represents a replication fork with the replisome (green) encountering RPII in a head-to-head configuration resulting in a potential transcription-replication conflict (TRC; right hand skull and crossed bones). It also shows a potential RPII-RPIII transcription-transcription conflict (TTC; left hand skull and crossed bones) caused by counter directional transcription of RNA polymerases. The letters and associated arrows indicate the possible pathways by which Drc1 and Tsn1 might interact with the macromolecules associated with RPII to mediate its dissociation with the DNA template. A–Direct Dcr1-RPII interaction; B–Direct Tsn1-RPII interaction; C–Tsn1 direct interaction with ssRNA of nascent transcript; D–Tsn1 direct interaction with dsRNA formed by intramolecular pairing in the nascent transcript; E–Tsn1 direct association with dsRNA formed by intermolecular association of nascent transcripts produced from opposite strands; F- Tsn1 direct interaction with ssDNA associated with the transcription bubble; G–Tsn1 indirect interaction with nascent transcript via binding to spliceosome-like factor Srp1.

It is possible that the RPII displacement mechanism is mediated by a direct protein-protein association of either Dcr1 or Tsn1 with RPII (Fig 6A and 6B, respectively). In support of this, Trax does provide a nuclear scaffold role for ATM in the DNA damage response via direct interaction, and whilst this is Translin-independent it demonstrates the capacity for the Translin super family to directly interact with other non-paralogous proteins during the DNA damage response [23], something that has also been observed for Trax interaction with the non-homologous end joining protein C1D [80]. Additionally, Translin can interact with other proteins (see below), including the DNA repair factor GADD34 [21].

It is tempting to speculate that Translin, and Dicer, function to displace RPII via binding to one of the RNAs associated with RPII at TRC or TTC sites (Fig 6); this could be nascent single-stranded transcript (Fig 6C), double-stranded RNA structures generated by nascent transcript intra-molecular base pairing (Fig 6D) or by inter-molecular base pairing between nascent transcripts generated from opposing template DNA strands (Fig 6E). Whilst *S*. *pombe* Tsn1 appears to favour RNA binding, it does have DNA binding capability [5,81] and single-stranded DNA generated at the transcriptional bubble or by the associated torsional stress could also be a substrate for Translin binding to target RPII displacement (Fig 6F). Notwithstanding this, it is not unreasonable to postulate that it is an RNA binding function of Translin that is require as this is consistent with its other known roles in RNA metabolism and mRNA binding, such as the direct binding to *BDNF* mRNA, where Translin binding defects can result in memory and psychiatric disorders [71].

Eliahoo and co-workers [82] identified the *S*. *pombe* pre-mRNA splicing factor Srp1 as a putative Tsn1 binding partner. Efficient cleavage of pre-mRNAs is known to prevent genomic instability associated with DNA replication stress [83] and introns protect genomes from replicative stress, indicating that some feature of spliceosome-associated processing is protective [84]. However, we observed increased recombination associated with the production of transcripts that have no discernable intronic sequences (Fig 4A). It is known that some spliceosome components have non-canonical roles in genome stability maintenance [85], so potentially Tsn1 functions in concert with the spliceosome-like Srp1 to act upon nascent transcripts in a splicing-independent fashion (Fig 6G). A role for spliceosome/spliceosome-like function in *S*. *pombe* is further supported by the finding that the spliceosome-associated protein Nrl1 is involved in suppressing R-loop formation and homologous recombination [86].

How might this connect Translin to chromosomal translocations? It has recently been demonstrated that cancer chromosomal translocations are linked to topoisomerase-mediated DSBs that are not associated with paused RPII *per se*, rather topoisomerase-induced DSBs are generated at sites from which paused RPII have been removed [87]. We propose a model in which Translin associates with pre-mRNA, possibly in conjunction with spliceosome factors, to assist the removal of paused RPII that triggers translocation-susceptible DSBs. This offers a plausible mechanism to address the longstanding question of why Translin associates with disease-linked translocation breakpoint junctions.

We have additionally revealed a possible functional redundancy between Rnh201 and Tsn1. RNase H activity is required for the removal of R-loops and the canonical view is that R-loops induce replicative stress and genome instability [41–46,88]. Originally, we had been working on the hypothesis that *S*. *pombe* Tsn1 might suppress R-loop levels and loss of Tsn1 function would further elevate potentially genotoxic levels of R-loops above the levels seen in following loss of Dcr1. However, this transpired not be the case. Surprisingly loss of both Tsn1 and Tfx1 elevate R-loops at tDNA and rDNA loci, to levels equal to those seen in the Dcr1-deficient cells. These levels of R-loops are not sufficient to increase genetic instability in replicative stress, indicating that R-loops *per se* are not overly problematic. Moreover, R-loop levels were not significantly increased in the *dcr1*Δ *tsn1*Δ double mutant relative to the *dcr1*Δ single mutant, despite there being an elevation in sensitivity to replicative stress and increased polar tDNA recombination. Whilst R-loops are elevated following loss of Tsn1 function in a Dcr1-proficient background, RPII is not retained at elevated levels indicting functional Dcr1 is sufficient for appropriate RPII displacement. This fits with the model describe above, in which Tsn1 functions to prevent further genomic stress by displacing RPII in Dcr1-deficiency. Interestingly, recent work using budding yeast led Zardoni and co-workers to suggest that it is RPII in active elongation mode is the major replicative obstacle which drives genome instability [89] and so it will be important to determine whether Tsn1 and/or Dcr1 displace RPII in elongation mode or a more static configuration.

This brings into question what causes enhanced replicative stress sensitivity in the *tsn1*Δ *rnh201*Δ background and suggests that this is not due to a failure to suppress R-loop levels, which is supported by the failure to suppress this phenotype by overexpressing *rnh1*^+^. We believe that these observations point to a potential additional function for Rnh201. Mis-incorporated ribonucleotide can retard replication [90], so the need for RNase H2 for their removal could impair replicative progression in the *rnh201*Δ mutant. When this is combined with HU-induced stress Tsn1 becomes required for RPII template dissociation, despite Dcr1 proficiency; that is to say, under the elevated replicative stress caused by loss of Rnh201 the Tsn1-mediated auxiliary pathway for RPII template dissociation is required.

However, in humans, RNase H2 has been proposed to also have non-enzymatic activities, this is partly based on the fact that mutations in the genes coding for the human RNASEH2B and RNASEH2C, which disrupt protein-protein interaction functions and not catalytic activity, are linked to Aicardi-Goutiéres syndrome [51]. Given this, it is possible that Rnh201 provides another, as yet undetermined, non-enzymatic function to suppress susceptibility to replicative stress.

Previously, only relatively limited evidence linked Translin to the mechanisms of chromosomal translocation formation. Here we provide the first mechanistic insight into how conserved Translin function might be linked to genomic changes such as translocations, drivers of evolution and disease. Importantly, given that Translin has been targeted as a potential therapeutic target in oncology and other disorders [13–15], these findings also provide insight to inform rational therapeutic design to ensure that only disease associated function(s) are targeted, and also exposes new synthetic lethality therapeutic options should it be found that Translin provides an auxiliary function in Dicer deficiency in human cells.

## Supporting information

S1 Table Strains used in this study. (DOCX)

S2 Table Primers used in this study. (DOCX)

S1 Fig Over expression of *S*. *pombe tsn1*^+^, *S*. *pombe dcr1*^+^ and human *TSN*^+^ suppress the elevated HU sensitivity of the *S*. *pombe dcr1*Δ *tsn1*Δ double mutant. Appropriate strains were spotted onto EMM media with (left hand pair) and without (right hand pair) thiamine [which suppresses gene overexpression (OE) from pREP3X *nmt* promoter] containing no HU or 10 mM HU. In the presence of thiamine the overexpression of either *S*. *pombe tsn1*^+^ (Sp*tsn1*^+^), *S*. *pombe dcr1*^+^ (Sp*dcr1*^+^) or human *TSN*^+^ (Hu*TSN*^+^) result in suppression of the elevated HU sensitivity of the *S*. *pombe dcr1*Δ *tsn1*Δ double mutant.
(PDF)

S2 Fig Over expression of *S*. *pombe tfx1*^+^ or human *TSNAX*^+^ cannot substitute for the loss of *S*. *pombe tsn1*+. 10-fold serial dilutions of indicated strains were spotted onto EMM with thiamine (with and without HU; 10 mM) or without thiamine (with or without HU; 10 mM). OE-Sp*tfxfx—*overexpressed *S*. *pombe tsn1*+; OE-Hu*TSNAX*+*—*overexpressed human *TSNAX*+; OE-Sp*rnh1*+—overexpressed *S*. *pombe rnh1*+.
(PDF)

S3 Fig *S*. *pombe rnh201*Δ mutants but not *rnh1*Δ mutants exhibit sensitivity to replicative stress induced by HU when in logarithmic proliferation, but not lag or stationary phase. Appropriate *S*. *pombe* strains were spotted out onto YEA without (left hand set) or with 10 mM HU (right hand set). The same cultures were spotted out from the three distinct phases of proliferative expansion, lag (top row), logarithmic (middle row) or stationary (bottom row). Only logarithmically proliferating *rnh201*Δ single mutants exhibited HU sensitivity. *rnh1*Δ single mutants exhibited no HU sensitivity for all culture stages.
(PDF)

S4 Fig Overexpression of RNases does not compensate for loss of Tsn1 or Dcr1 function. (A) RT-qPCR analysis of expression of *rnh1*^+^ in distinct strains. pREP3X is the empty vector control. Normalization was carried out against *act1*^+^ levels. Error bars represent standard deviation (triplicate biological repeats). (B) RT-qPCR analysis of expression of *pac1*^+^ in distinct strains. pREP3X is the empty vector control. Normalization was carried out against *act1*^+^ levels. Error bars represent standard deviation (triplicate biological repeats). (C) Overexpression of *rnh201*^+^ does not suppress requirement for Tsn1 to tolerate HU in the absence of Dcr1. OE = overexpression. In the presence of thiamine *rnh201*^+^ is not overexpressed from the vector (pREP3X); overexpression is triggered upon the removal of thiamine. Overexpression of *rnh201*^+^ in the absence of thiamine suppresses the HU sensitivity phenotype of the *rnh1*Δ *rnh201*Δ double mutant, which demonstrates the *rnh201*^+^ overexpression is active on these plates. (D) Overexpression of *rnh1*^+^ or *pac1*^+^ does not suppress requirement for Dcr1 to tolerate HU. OE = overexpression. In the presence of thiamine *rnh1*^+^ and *pac1*^+^ are not overexpressed from the vector (pREP3X). Overexpression of *rnh1*^+^ in the absence of thiamine suppresses the HU sensitivity phenotype of the *rnh1*Δ *rnh201*Δ double mutant, which demonstrates the *rnh201*^+^ overexpression is active on these plates.
(PDF)

S5 Fig Mutation of the conserved tsn1+ residue does not diminish DNA replicative stress response function. (A) Alignment of human and *S*. *pombe* Translin (Hu TSN and Sp Tsn1, respectively) and Trax (Hu TSNAX and Sp Tfx1, respectively) paralogues. Amino acids highlighted in blue are required for Trax RNase activity and are not conserved in Translin. The residue marked in red (E197) is an RNase catalytic residue for human TSNAX (Trax) which is the only RNase catalytic residue to be conserved in both species. Amino acid residue numerical designations refer to the human TSNAX sequence. (B) Mutation of the conserved RNase catalytic residue in *S*. *pombe tsn1* does not disrupt genome stability function. Over expression (OE) of the *S*. *pombe tsn1-E152A* mutant allele can suppress the loss of *tsn1* function to the same degree as the *tsn1*^+^ wild-type control.
(PDF)

S6 Fig *tsn1*Δ and *tfx1*Δ single mutants have increased RNA:DNA hybrids at *tDNAHIS*. Quantification of DRIP for the *tDNAHIS* locus for the indicated strains. *P>0.05, **P>0.01, ***P>0.001, ns–not significant, from *t*-test in pairwise comparisons relative to relative to wild-type (black) or to *dcr1*Δ (red). Bars represent standard deviation. Values are obtained from a minimum of three independent biological repeats.
(PDF)

S7 Fig Tsn1 is required to mediate displacement of RPII at the *rDNA* locus during Dcr1-deficiency. Quantification of RPII ChIP for the *rDNA* locus for the indicated strains. *P>0.05, **P>0.01, ***P>0.001, ns–not significant, relative to the wild-type (black) or to *dcr1*Δ (red). Bars represent standard deviation. Values are obtained from a minimum of three independent biological repeats.
(PDF)

## References

[1] Siri SM, Martion M, Gottifredi V. Structural chromosome instability: types, origins, consequences, and therapeutic opportunities. Cancers. 2021; 13: 3056. doi: 10.3390/cancers13123056 34205328 PMC8234978

[2] MacDonald C, McClelland SE. Chromosome instability through the ages: parallels between speciation and somatic (cancer) evolution. Trends Genet. 2021; 37: 691–694. doi: 10.1016/j.tig.2021.05.003 34083067

[3] Dahiya R, Hu Q, Ly P. Mechanistic origins of diverse genome rearrangements in cancer. Semin. Cell Dev. Biol. 2021; 123: 100–109. doi: 10.1016/j.semcdb.2021.03.003 33824062 PMC8487437

[4] Aoki K, Suzuki K, Sugano T, Tasaka T, Nakahara K, Kuge O, et al. A novel gene, Translin, encodes a recombination hotspot binding protein associated with chromosomal translocations. Nat. Genet. 1995; 10: 167–174. doi: 10.1038/ng0695-167 7663511

[5] Jaendling A, McFarlane RJ. Biological roles of translin and trax-associated factor X: RNA metabolism comes to the fore. Biochem. J. 2010; 429: 225–234. doi: 10.1042/BJ20100273 20578993

[6] Gupta A, Pillai VS, Chittela RK. Translin: a multifunctional protein involved in nucleic acid metabolism. J. Biosci. 2019; 44: 139. 10.1007/s12038-019-9947-6 31894120

[7] Liu Y, Ye X, Jiang F, Liang C, Chen D, Peng J, et al. C3PO, an endoribonuclease that promotes RNAi by facilitating RISC activation. Science. 2009; 325: 750–753. doi: 10.1126/science.1176325 19661431 PMC2855623

[8] Ye X, Huang N, Liu Y, Paroo Z, Huerta C, Li P, et al. Structure of C3PO and mechanism of human RISC activation. Nat. Struct. Mol. Biol. 2011; 18: 650–657. doi: 10.1038/nsmb.2032 21552258 PMC3109212

[9] Tian Y, Simanshu DK, Ascano M, Diaz-Avalos R, Park AY, Juranek SA, et al. Multimeric assembly and biochemical characterization of the Trax-translin endonuclease complex. Nat. Struct. Mol. Biol. 2011; 18: 658–664. doi: 10.1038/nsmb.2069 21552261 PMC3109869

[10] Li L, Gu W, Liang C, Liu Q, Mello CC, Liu Y. The translin-TRAX complex (C3PO) is a ribonuclease in tRNA processing. Nat. Struct. Mol. Biol. 2012; 19: 824–830. doi: 10.1038/nsmb.2337 22773104 PMC3414638

[11] Baraban JM, Shah A, Fu X. Multiple pathways mediate microRNA degradation: focus on the Translin/Trax RNase complex. Adv. Pharmacol. 2018; 82: 1–20. doi: 10.1016/bs.apha.2017.08.003 29413516

[12] Li Z, Wu Y, Baraban JM. The Translin/Trax RNA binding complex: clues to function in the nervous system. Biochim. Biophys. Acta. 2008; 1779: 479–485. doi: 10.1016/j.bbagrm.2008.03.008 18424275 PMC2561206

[13] McFarlane RJ, Wakeman JA. Translin-Trax: considerations for oncological therapeutic targeting. Trends Cancer. 2020; 6: 450–453. doi: 10.1016/j.trecan.2020.02.014 32460000

[14] Asada K, Canestrari E, Fu X, Li Z, Makowski E, Wu Y-C, et al. Rescuing dicer defects via inhibition of an anti-dicing nuclease. Cell Rep. 2014; 9: 1471–1481. doi: 10.1016/j.celrep.2014.10.021 25457613 PMC4303555

[15] Asada K, Canestrari E, Paroo Z. A druggable target for rescuing microRNA defects. Bioorg. Med. Chem. Lett. 2016; 26: 4942–4946. doi: 10.1016/j.bmcl.2016.09.019 27641467

[16] Tuday E, Nomura Y, Ruhela D, Nakano M, Fu X, Shah A, et al. Deletion of the microRNA-degrading nuclease, translin-trax, prevents pathogenic vascular stiffness. Am. J. Physiol. Heart Circ. Physiol. 2019; 317: H1116–H1124. doi: 10.1152/ajpheart.00153.2019 31625778 PMC6879913

[17] Tuday E, Nakano M, Akiyoshi K, Fu X, Shah AP, Yamaguchi A, et al. Degradation of premature-miR-181b by the Translin/Trax RNase increases vascular smooth muscle cell stiffness. Hypertension. 2021; 78: 831–839. doi: 10.1161/HYPERTENSIONAHA.120.16690 34304585 PMC8363557

[18] Geiger R, Rieckmann JC, Wolf T, Basso C, Feng Y, Fuhrer T, et al. L-Arginine modulates T cell metabolism and enhances survival and anti-tumor activity. Cell. 2016; 167: 829–842. doi: 10.1016/j.cell.2016.09.031 27745970 PMC5075284

[19] Kasai M, Matsuzaki T, Katayanagi K, Omori A, Maziarz RT, Strominger JL, et al. The translin ring specifically recognizes DNA ends at recombination hot spots in the human genome. J. Biol. Chem. 1997; 272: 11402–11407. doi: 10.1074/jbc.272.17.11402 9111049

[20] Fukuda K, Ishida R, Aoki K, Nakahara K, Takashi T, Mochida K, et al. Contribution of Translin to hematopoietic regeneration after sublethal ionizing irradiation. Biol. Pharm. Bull. 2008; 31: 207–211. doi: 10.1248/bpb.31.207 18239274

[21] Hasegawa T, Isobe K. Evidence for the interaction between Translin and GADD34 in mammalian cells. Biochim. Biophys. Acta. 1999; 1428: 161–168. doi: 10.1016/s0304-4165(99)00060-4 10434033

[22] Koh M, Ahmad I, Ko Y, Zhang Y, Martinez TF, Diedrich JK, et al. A short ORF-encoded transcriptional regulator. Proc. Natl. Acad. Sci. USA 2021; 118: e2021943118. doi: 10.1073/pnas.2021943118 33468658 PMC7848545

[23] Wang J-Y, Chen S-Y, Sun C-N, Chien T, Chern Y. A central role of TRAX in the ATM-mediated DNA repair. Oncogene. 2016; 35: 1657–1670. doi: 10.1038/onc.2015.228 26096928

[24] Forsburg SL, Rhind N. Basic methods for fission yeast. Yeast. 2006; 23: 173–183. doi: 10.1002/yea.1347 16498704 PMC5074380

[25] Sabatinos SA, Forsburg SL. Molecular genetics of *Schizosaccharomyces pombe*. Methods Enzymol. 2010; 470: 759–795. doi: 10.1016/S0076-6879(10)70032-X 20946835

[26] Bähler J, Wu JQ, Longtine MS, Shah NG, McKenzie 3^rd^ A, Steever AB, et al. Heterologous modules for efficient and versatile PCR-based gene targeting in *Schizosaccharomyces pombe*. Yeast. 1998; 14: 943–951. 10.1002/(sici)1097-0061(199807)14:10<943::aid-yea292>3.0.co;2-y 9717240

[27] Niwa O, Matsumoto T, Yanagida M. Construction of a mini-chromosome by deletion and its mitotic and meiotic behaviour in fission yeast. Mol. Gen. Genet. 1986; 203: 397–405. 10.1007/BF00422063

[28] Niwa O, Matsumoto T, Chikashige Y, Yanagida M. Characterization of Schizosaccharomyces pombe minichromosome deletion derivatives and a functional allocation of their centromere. EMBO J. 1989; 8: 3045–3052. doi: 10.1002/j.1460-2075.1989.tb08455.x 2583093 PMC401382

[29] Niwa O. Determination of the frequency of minichromsome loss to assess chromosome stability in fission yeast. Cold Spring Harb. Protoc. 2018; 2018: 1–4. doi: 10.1101/pdb.prot091991 27343268

[30] Pryce DW, Ramayah S, Jaendling A, McFarlane RJ. Recombination at DNA replication fork barriers is not universal and is differentially regulated by Swi1. Proc. Natl. Acad. Sci. USA 2009; 106: 4770–4775. doi: 10.1073/pnas.0807739106 19273851 PMC2660728

[31] Pryce DW, Lorenz A, Smirnova JB, Loidl J, McFarlane RJ. Differential activation of M26-containing meiotic recombination hot spots in *Schizosaccharomyces pombe*. Genetics. 2005; 170: 95–106. doi: 10.1534/genetics.104.036301 15744055 PMC1449712

[32] Forsburg SL. Comparison of *Schizosaccharomyces pombe* expression systems. Nucleic Acids Res. 1993; 21: 2955–2956. doi: 10.1093/nar/21.12.2955 8332516 PMC309706

[33] Maundrell K. Thiamine-repressible expression vectors pREP and pRIP for fission yeast. Gene. 1993; 123: 127–130. doi: 10.1016/0378-1119(93)90551-d 8422996

[34] McFarlane R. Translin facilitates RNA polymerase II dissociation and suppresses genome instability during RNase H2- and Dicer-deficiency (data files). 2022. Dryad, Dataset, 10.5061/dryad.n5tb2rbx8. PMC9246224 35714159

[35] Jaendling A, Ramayah S, Pryce DW, McFarlane RJ. Functional characterization of the *Schizosaccharomyces pombe* homologue of the leukaemia-associated translocation breakpoint junction binding protein translin and its binding partner, TRAX. Biochim. Biophys. Acta. 2008; 1783: 203–213. doi: 10.1016/j.bbamcr.2007.10.014 18062930

[36] Gomez-Escobar N, Almobadel N, Alzahrani O, Feichtinger J, Planells-Palop V, Alshehri Z, et al. Translin and Trax differentially regulate telomere-associated transcript homeostasis. Oncotarget. 2016; 7: 33809–33820. doi: 10.18632/oncotarget.9278 27183912 PMC5085120

[37] Zaratiegui M, Castel SE, Irvine DV, Kloc A, Ren J, Li F, et al. RNAi promotes heterochromatic silencing through replication-coupled release of RNA Pol II. Nature. 2011; 479: 135–138. doi: 10.1038/nature10501 22002604 PMC3391703

[38] Castel SE, Ren J, Bhattacharjee S, Chang A-Y, Sánchez M, Valbuena A, et al. Dicer promotes transcription termination at sites of replication stress to maintain genome stability. Cell. 2014; 159: 572–583. doi: 10.1016/j.cell.2014.09.031 25417108 PMC4243041

[39] Ren J, Castel SE, Martienssen RA. Dicer in action at replication-transcription collisions. *Mol*. Cell. Oncol. 2015; 2: e991224. doi: 10.4161/23723556.2014.991224 27308471 PMC4905307

[40] Crossley MP, Bocek M, Cimprich KA. R-loops as cellular regulators and genomic threats. Mol. Cell. 2019; 73: 398–411. doi: 10.1016/j.molcel.2019.01.024 30735654 PMC6402819

[41] García-Muse T, Aguilera A. R loops: from physiological to pathological roles. Cell. 2019; 179: 604–618. doi: 10.1016/j.cell.2019.08.055 31607512

[42] Wells JP, White J, Stirling PC. R loops and their composite cancer connections. Trends Cancer. 2019; 5: 619–631. doi: 10.1016/j.trecan.2019.08.006 31706509

[43] Brambati A, Zardoni L, Nardini E, Pellicioli A, Liberi G. The dark side of RNA:DNA hybrids. Mutat. Res. 2020; 784: 108300. doi: 10.1016/j.mrrev.2020.108300 32430097

[44] Hegazy YA, Fernando CM, Tran EJ. The balancing act of R-loop biology: the good, the bad, and the ugly. J. Biol. Chem. 2020; 295: 905–913. doi: 10.1074/jbc.REV119.011353 31843970 PMC6983857

[45] Nierhs C, Luke B. Regulatory R-loops as facilitators of gene expression and genome stability. Nat. Rev. Mol. Cell Biol. 20202; 21: 167–178. doi: 10.1038/s41580-019-0206-3 32005969 PMC7116639

[46] Rinaldi C, Pizzul P, Longhese MP, Bonetti D. Sensing R-loop-associated DNA damage to safeguard genome stability. Front. Cell Dev. Biol. 2021; 8: 618157. doi: 10.3389/fcell.2020.618157 33505970 PMC7829580

[47] Michelini F, Pitchiaya S, Vitelli V, Sharma S, Gioia U, Pessina F, et al. Damage-induced lncRNAs control the DNA damage response through interaction with DDRNAs at individual double-strand breaks. Nat. Cell Biol. 2017; 19: 1400–1411. doi: 10.1038/ncb3643 29180822 PMC5714282

[48] Liu S, Hua Y, Wang J, Li L, Yuan J, Zhang B, et al. RNA polymerase III is required for the repair of DNA double-strand breaks by homologous recombination. Cell. 2021; 184: 1314–1329. doi: 10.1016/j.cell.2021.01.048 33626331

[49] Pessina F, Gioia U, Brandi O, Farina S, Ceccon M, Francia S, d’Adda di Fagagna F. DNA damage triggers a new phase in neurodegeneration. Trends Genet. 2021; 37: 337–354. doi: 10.1016/j.tig.2020.09.006 33020022

[50] Hyjek M, Figeil M, Nowotny M. RNase H: structure and mechanism. DNA Repair. 2019; 84: 102672. doi: 10.1016/j.dnarep.2019.102672 31371183

[51] Feng S, Cao Z. Is the role of human RNase H2 restricted to its enzyme activity? Prog. Biophys. Mol. Biol. 2016; 121: 66–73. doi: 10.1016/j.pbiomolbio.2015.11.001 26603688

[52] Ohle C, Tesorero R, Schermann G, Dobrev N, Sinning I, Fischer T. Transient RNA-DNA hybrids are required for efficient double-strand break repair. Cell. 2016; 167: 1001–1013. doi: 10.1016/j.cell.2016.10.001 27881299

[53] Zhao H, Zhu M, Limbo O, Russell P. RNase H eliminates R-loops that disrupt DNA replication but is nonessential for efficient DSB repair. EMBO Rep. 2018; 19: e45335. doi: 10.15252/embr.201745335 29622660 PMC5934767

[54] Zimmer AD, Koshland D. Differential roles of the RNases H in preventing chromosome instability. Proc. Natl. Acad. Sci. USA. 2016; 113: 12220–12225. doi: 10.1073/pnas.1613448113 27791008 PMC5086985

[55] Lockhart A, Borges Pires V, Bento F, Kelloner V, Luke-Glaser S, Yakoub G, et al. RNase H1 and H2 are differentially regulated to process RNA-DNA hybrids. Cell Rep. 2019; 29: 2890–2900. doi: 10.1016/j.celrep.2019.10.108 31775053

[56] Wang J, Boja ES, Oubrahim H, Chock PB. Testis brain ribonucleic acid-binding protein/translin possesses both single-stranded and double-stranded ribonuclease activities. Biochemistry. 2004; 43: 13424–13431. doi: 10.1021/bi048847l 15491149

[57] Eliahoo E, Ben Yosef R, Pérez-Cano L, Fernández-Recio J, Glaser F, Manor H. Mapping of interaction sites of the *Schizosaccharomyces pombe* protein Translin with nucleic acids and proteins: a combined molecular genetics and bioinformatics study. Nucleic Acids Res. 2010; 38: 2975–2989. doi: 10.1093/nar/gkp1230 20081200 PMC2875027

[58] Parizotto EA, Lowe ED, Parker JS. Structural basis for duplex RNA recognition and cleavage by *Archaeoglobus fulgidus* C3PO. Nat. Struct. Mol. Biol. 2013; 20: 380–386. doi: 10.1038/nsmb.2487 23353787 PMC3597040

[59] Luke B, Panza A, Redon S, Iglesias N, Li, Linger J. The Rat1p 5’ to 3’ exonuclease degrades telomeric repeat-containing RNA and promotes telomere elongation in *Schaccharomyces cerevisiae*. Mol. Cell. 2008; 32: 465–477. doi: 10.1016/j.molcel.2008.10.019 19026778

[60] Hartono SR, Malapert A, Legros P, Bernard P, Chédin F, Vanoosthuyse V. The affinity of the S9.6 antibody for double-stranded RNAs impacts the accurate mapping of R-loops in fission yeast. J. Mol. Biol. 2018; 430: 272–284. doi: 10.1016/j.jmb.2017.12.016 29289567 PMC5987549

[61] Begnis M, Apte MS, Masuda H, Jain D, Wheeler DL, Cooper JP. RNAi drives nonreciprocal translocations at eroding chromosome ends to establish telomere-free linear chromosomes. Genes Dev. 2018; 32: 537–554. doi: 10.1101/gad.311712.118 29654060 PMC5959237

[62] Jain D, Hebden AK, Nakamura TM, Miller KM, Cooper JP. HAATI survivors replace canonical telomeres with blocks of generic heterochromatin. Nature. 2010; 467: 223–227. doi: 10.1038/nature09374 20829796

[63] Tan-Wong SM, Dhir S, Proudfoot NJ. R-loops promote antisense transcription across the mammalian genome. Mol. Cell. 2019; 76: 600–616. doi: 10.1016/j.molcel.2019.10.002 31679819 PMC6868509

[64] McFarlane RJ, Whitehall SK. tRNA genes in eukaryotic genome organization and reorganization. Cell Cycle. 2009; 8: 3102–3106. doi: 10.4161/cc.8.19.9625 19738425

[65] Guimarães AR, Correia I, Sousa I, Oliveira C, Moura G, Bezerra AR, et al. tRNAs as a driving force of genome evolution in yeast. Front. Microbiol. 2021; 12: 634004. doi: 10.3389/fmicb.2021.634004 33776966 PMC7990762

[66] Steinacher R, Osman F, Dalgaard JZ, Lorenz A, Whitby MC. The DNA helicase Pfh1 promotes fork merging at replication termination sites to ensure genome stability. Genes Dev. 2012; 26: 594–602. doi: 10.1101/gad.184663.111 22426535 PMC3315120

[67] Jalan M, Oehler J, Morrow CA, Osman F, Whitby MC. Factors affecting template switch recombination associated with restarted DNA replication. Elife. 2019; 8: e41697. doi: 10.7554/eLife.41697 30667359 PMC6358216

[68] Yang S, Cho YS, Chennathukuzhi VM, Underkoffler LA, Loomes K, Hecht NB. Translin-associated factor X is post-transcriptionally regulated by its partner protein TB-RBP, and both are essential for normal cell proliferation. J. Biol. Chem. 2004; 279: 12605–12614. doi: 10.1074/jbc.M313133200 14711818

[69] Han JR, Gu W, Hecht NB. Testis-brain RNA binding protein, a testicular translational regulatory RNA-binding protein, is present in the brain and binds to the 3’ untranslated regions of transported brain mRNAs. Biol. Reprod. 1995; 53: 707–717. doi: 10.1095/biolreprod53.3.707 7578697

[70] Wu XQ, Gu W, Meng X, Hecht NB. The RNMA-binding protein, TB-RBP, is the mouse homologue of translin, a recombination protein associated with chromosomal translocations. Proc. Natl. Acad. Sci. USA. 1997; 94: 5640–5645. doi: 10.1073/pnas.94.11.5640 9159125 PMC20831

[71] Chiaruttini C, Vicario A, Li Z, Baj G, Braiuca P, Wu Y, et al. Dendritic trafficking of BDNF mRNA is mediated by translin and blocked by the G196A (Val66Met) mutation. Proc. Natl. Acad. Sci. USA. 2009; 106: 16581–16486. doi: 10.1073/pnas.0902833106 19805324 PMC2752540

[72] Murakami K, Yurgel ME, Stahl BA, Masek P, Mehta A, Heidker R, et al. Translin is required for metabolic regulation of sleep. Curr. Biol. 2016; 26: 972–980. doi: 10.1016/j.cub.2016.02.013 27020744 PMC4846466

[73] Park AJ, Shetty MS, Baraban JM, Abel T. Selective role of the translin/trax RNase complex in hippocampal synaptic plasticity. Mol. Brain. 2020; 13: 145. doi: 10.1186/s13041-020-00691-5 33172471 PMC7653721

[74] Shah AP, Johnson MD, Fu X, Boersma GJ, Shah M, Wolfgang MJ, et al. Deletion of translin (Tsn) induces robust adiposity and hepatic steatosis without impairing glucose tolerance. Int. J. Obes (Lond). 2020; 44: 254–266. doi: 10.1038/s41366-018-0315-7 30647452 PMC6629527

[75] Kumar MS, Pester RE, Chen CY, Lane K, Chin C, Lu J, et al. Dicer1 functions as a haploinsufficient tumor suppressor. Genes Dev. 2009; 23: 2700–2704. doi: 10.1101/gad.1848209 19903759 PMC2788328

[76] Vedanayagam J, Chatila WK, Arman Aksoy B, Majumdar S, Jacobsen Skanderup A, Demir E, et al. Cancer-associated mutations in DICER1 RNase IIIa and IIIb domains exerts similar effects on miRNA biogenesis. Nat. Commun. 2019; 10: 3682. doi: 10.1038/s41467-019-11610-1 31417090 PMC6695490

[77] Francia S, Michelini F, Saxena A, Tang D, de Hoon M, Anelli V, et al. Site-specific DICER and DROSHA RNA products control the DNA-damage response. Nature. 2012; 488: 231–235. doi: 10.1038/nature11179 22722852 PMC3442236

[78] Burger K, Schlackow M, Potts M, Hester S, Mohammed S, Gullerova M. Nuclear phosphorylated Diver processes double-stranded RNA in response to DNA damage. J. Cell Biol. 2017; 216: 2373–2389. doi: 10.1083/jcb.201612131 28642363 PMC5551710

[79] Fragkos M, Barra V, Egger T, Bordignon B, Lemacon D, Naim V, and Coquelle A, et al. Dicer prevents genome instability in response to replication stress. Oncotarget. 2019; 10: 4407–4423. doi: 10.18632/oncotarget.27034 31320994 PMC6633883

[80] Erdemir T, Bilican B, Oncel D, Goding CR, Yavuzer U. DNA damage-dependent interaction of the nuclear matrix protein C1D with Translin-associated factor X (TRAX). J. Cell Sci. 2002; 115: 207–216. doi: 10.1242/jcs.115.1.207 11801738

[81] Laufman O, Yosef RB, Adir N, Manor H. Cloning and characterization of the *Schizosaccharomyces pombe* homologs of the human protein Translin and Translin-associated protein TRAX. Nucleic Acids Res.2005; 33: 4128–4139. doi: 10.1093/nar/gki727 16043634 PMC1180670

[82] Eliahoo E, Litovco P, Ben Yosef R, Bendalak K, Ziv T, Manor H. Identification of proteins that form specific complexes with the highly conserved protein Translin in *Schizosaccharomyces pombe*. Biochim. Biophys. Acta. 2014; 1844: 767–777. doi: 10.1016/j.bbapap.2013.12.016 24382491

[83] Teloni F, Michelena J, Lezaja A, Kilic S, Ambrosi C, Menon S, et al. Efficient pre-mRNA cleavage prevents replication-stress-associated genome instability. Mol. Cell. 2019; 73: 670–683. doi: 10.1016/j.molcel.2018.11.036 30639241 PMC6395949

[84] Bonnet A, Grosso AR, Elkaoutari A, Coleno E, Presle A, Sridhara AC, et al. Introns protect eukaryotic genomes from transcription-associated genetic instability. Mol. Cell. 2017; 67: 608–621. doi: 10.1016/j.molcel.2017.07.002 28757210

[85] Tam AS, Stirling PC. Splicing, genome stability and disease: splice like your genome depends on it! Curr. Genet. 2019; 65: 905–912. doi: 10.1007/s00294-019-00964-0 30953124

[86] Aronica L, Kasparek T, Ruchman D, Maequez Y, Cipak L, Cipakova I, et al. The spliceosome-associated protein Nrl1 suppresses homologous recombination-dependent R-loop formation in fission yeast. Nucleic Acids Res. 2016; 44: 1703–1717. doi: 10.1093/nar/gkv1473 26682798 PMC4770224

[87] Dellino GI, Palluzzi F, Chiariello AM, Piccioni R, Bianco S, Furia L, et al. Release of paused RNA polymerase II at specific loci favors double-strand-break formation and promotes cancer translocations. Nat. Genet. 2019; 51: 1011–1023. doi: 10.1038/s41588-019-0421-z 31110352

[88] Gómez-González B, Aguilera A. Transcription-mediated replication hinderance: a major driver of genomic instability. Genes Dev. 2019; 33: 1008–1026. doi: 10.1101/gad.324517.119 31123061 PMC6672053

[89] Zardoni L, Nardini E, Brambati A, Lucca C, Choudhary R, Loperfido F, et al. Elongating RNA polymerase II and RNA:DNA hybrids hinder fork progression and gene expression at sites of head-on replication-transcription collisions. Nucleic Acids Res. 2021; 49: 12769–12784. doi: 10.1093/nar/gkab1146 34878142 PMC8682787

[90] Watt DL, Johansson E, Burgers PM, Kunkel TA. Replication of ribonucleotide-containing DNA templates by yeast replicative polymerases. DNA Repair. 2011; 10: 897–902. doi: 10.1016/j.dnarep.2011.05.009 21703943 PMC3147116

