# Supplementary material for: Translin facilitates RNA polymerase II dissociation and suppresses genome instability during RNase H2- and Dicer-deficiency
Source: PLoS Genet. 2022 Jun 17;18(6):e1010267. doi: 10.1371/journal.pgen.1010267 (PMC9246224; doi:10.1371/journal.pgen.1010267)
Supplement: S1 Table — (DOCX) [file pgen.1010267.s001.docx]

**S1 Table.** Strains used in this study

| **Strain** | **Genotype** | **Source** |
| --- | --- | --- |
|  |  |  |
| BP90 | *hˉ ade6-M26 ura4-D18 leu1-32* | McFarlane Collection |
| BP118 | *hˉ ade6-M216 ura4-D18 leu1-32 taz1::ura4*^+^ | McFarlane Collection |
| BP743 | *hˉ rad3-136* | McFarlane Collection |
| BP1080 | *hˉ ade6-M26 ura4-D18 leu1-32 tsn1::kanMX6* | McFarlane Collection |
| BP1089 | *hˉ ade6-M26 ura4-D18 leu1-32 tfx1::kanMX6* | McFarlane Collection |
| BP1534 | *hˉ ura4-D18 leu1-32 his3-D1 ade6::tRNA^GLU^ (1) lys1-37* (pSRS5) | McFarlane Collection |
| BP1535 | *hˉ ura4-D18 leu1-32 his3-D1 ade6::tRNA^GLU^ (2) lys1-37* (pSRS5) | McFarlane Collection |
| BP2294 | *hˉ ade6-M210 ura4-D18 leu1-32* Ch^16-23R^ | This study |
| BP2406 | *hˉ ade6-M210 ura4-D18 leu1-32 tfx1::kanMX6* Ch^16-23R^ | This study |
| BP2421 | *hˉ ade6-M210 ura4-D18 leu1-32 tsn1::kanMX6* Ch^16-23R^ | This study |
| BP2747 | *hˉ ade6-M26 ura4-D18 leu1-32 dcr1::natMX6* | McFarlane Collection |
| BP2748 | *hˉ ade6-M26 ura4-D18 leu1-32 dcr1::natMX6 tsn1::kanMX6* | McFarlane Collection |
| BP2750 | *hˉ ade6-M26 ura4-D18 leu1-32 dcr1::natMX6 tfx1::kanMX6* | McFarlane Collection |
| BP2757 | *hˉ ade6-M26 ura4-D18 leu1-32 ago1::ura4*^+^ | McFarlane Collection |
| BP2759 | *hˉ ade6-M26 ura4-D18 leu1-32 tsn1::kanMX6 ago1::ura4*^+^ | McFarlane Collection |
| BP2761 | *hˉ ade6-M26 ura4-D18 leu1-32 tfx1::kanMX6 ago1::ura4*^+^ | McFarlane Collection |
| BP2894 | *hˉ ade6-M210 ura4-D18 leu1-32 dcr1::ura4*^+^ Ch^16-23R^ | This study |
| BP2897 | *hˉ ade6-M210 ura4-D18 leu1-32 tfx1::kanMX6 dcr1::ura4*^+^ Ch^16-23R^ | This study |
| BP2899 | *hˉ ade6-M210 ura4-D18 leu1-32 tsn1::kanMX6 dcr1::ura4*^+^ Ch^16-23R^ | This study |
| BP3301 | *hˉ ade6-M210 ura4-D18 leu1-32 his3-D1* O*trt1::his3*^+^ | McFarlane Collection |
| BP3305 | *h+ ade6-M210 leu1-32 his3-D1 trt1::his3*^+^ (HAATI^STE^) | J. Cooper^1^ |
| BP3322 | *hˉ ura4-D18 leu1-32 his3-D1 ade6::tRNA^GLU^ (1) lys1-37 tsn1::kanMX6* (pSRS5) | McFarlane Collection |
| BP3324 | *hˉ ura4-D18 leu1-32 his3-D1 ade6::tRNA^GLU^ (1) lys1-37 dcr1::natMX6* (pSRS5) | McFarlane Collection |
| BP3326 | *hˉ ura4-D18 leu1-32 his3-D1 ade6::tRNA^GLU^ (1) lys1-37 dcr1::natMX6 tsn1::kanMX6* (pSRS5) | McFarlane Collection |
| BP3344 | *hˉ ura4-D18 leu1-32 his3-D1 ade6::tRNA^GLU^(2) lys1-37 tsn1::kanMX6* (pSRS5) | McFarlane Collection |
| BP3348 | *hˉ ura4-D18 leu1-32 his3-D1 ade6::tRNA^GLU^(2) lys1-37 dcr1::kanMX6* (pSRS5) | McFarlane Collection |
| BP3364 | *hˉ ura4-D18 leu1-32 his3-D1 ade6::tRNA^GLU^(2) lys1-37 tsn1::kanMX6 dcr1::natMX6* (pSRS5) | McFarlane Collection |
| BP3382 | *h+ ade6-M210 leu1-32 his3-D1 trt1::his3*^+^ (HAATI^STE^) *tsn1::kanMX6* | This study |
| BP3384 | *h+ ade6-M210 leu1-32 his3-D1 trt1::his3*^+^ (HAATI^STE^) *tfx1::kanMX6* | This study |
| BP3401 | *hˉ ade6-M26 ura4-D18 leu1-32 rnh1::kanMX6* | This study |
| BP3405 | *hˉ ade6-M26 ura4-D18 leu1-32 rnh201::kanMX6* | This study |
| BP3410 | *hˉ ade6-M26 ura4-D18 leu1-32 rnh1::kanMX6 rnh201::hphMX6* | This study |
| BP3412 | *hˉ ade6-M26 ura4-D18 leu1-32 tfx1::natMX6 rnh1::kanMX6* | This study |
| BP3414 | *hˉ ade6-M26 ura4-D18 leu1-32 tfx1::natMX6 rnh201::kanMX6* | This study |
| BP3417 | *hˉ ade6-M26 ura4-D18 leu1-32 rnh201::kanMX6 tsn1::natMX6* | This study |
| BP3426 | *hˉ ade6-M26 ura4-D18 leu1-32 tsn1::kanMX6 rnh1::natMX6* | This study |
| BP3428 | *hˉ ura4-D18 leu1-32 his3 ade6::tRNA^GLU^ (1) lys1-37 tfx1::kanMX6* (pSRS5) | McFarlane Collection |
| BP3431 | *hˉ ura4-D18 leu1-32 his3 ade6::tRNA^GLU^ (2) lys1-37 tfx1::kanMX6* (pSRS5) | McFarlane Collection |
| BP3433 | *hˉ ura4-D18 leu1-32 his3-D1 ade6::tRNA^GLU^ (1) lys1-37 dcr1::natMX6 tfx1::kanMX6* (pSRS5) | McFarlane Collection |
| BP3435 | *hˉ ura4-D18 leu1-32 his3 ade6::tRNA^GLU^(2) lys-37 dcr1::natMX6 tfx1::kanMX6* (pSRS5) | McFarlane Collection |
| BP3450 | *h+ ade6-M210 leu1-32 his3-D1 trt1::his3*^+^ (HAATI^STE^) *tsn1::kanMX6 rnh201::natMX6* | This study |
| BP3451 | *h+ ade6-M210 leu1-32 his3-D1 trt1::his3*^+^ (HAATI^STE^) *tsn1::kanMX6 rnh1::natMX6* | This study |
| BP3453 | *h+ ade6-M210 leu1-32 his3-D1 trt1::his3*^+^ (HAATI^STE^) *rnh201::natMX6* | This study |
| BP3454 | *h+ ade6-M210 leu1-32 his3-D1 trt1::his3*^+^ (HAATI^STE^) *rnh1::natMX6* | This study |
| BP3456 | *h+ ade6-M210 leu1-32 his3-D1 trt1::his3*^+^ (HAATI^STE^) *rnh201::natMX6 tfx1::kanMX6* | This study |
| BP3458 | *h+ ade6-M210 leu1-32 his3-D1 trt1::his3*^+^ (HAATI^STE^) *rnh1::natMX6 tfx1::kanMX6* | This study |
| BP3459 | *h+ ade6-M210 leu1-32 his3-D1 trt1::his3*^+^ (HAATI^STE^) *rnh201::natMX6 rnh1::kanMX6* | This study |
| BP3461 | *hˉ ade6-M26 ura4-D18 leu1-32 rnh201::kanMX6 dcr1::natMX6* | This study |
| BP3472 | *hˉ ade6-M26 ura4-D18 leu1-32 rnh1::kanMX6 rnh201::hphMX6* (pREP3X) | This study |
| BP3473 | *hˉ ade6-M26 ura4-D18 leu1-32 rnh1::kanMX6 rnh201::hphMX6* (pREP3X-*rnh1*^+^) | This study |
| BP3474 | *hˉ ade6-M26 ura4-D18 leu1-32 dcr1::natMX6 tsn1::kanMX6* (pREP3X) | This study |
| BP3475 | *hˉ ade6-M26 ura4-D18 leu1-32 dcr1::natMX6 tsn1::kanMX6* (pREP3X-*rnh1*^+^) | This study |
| BP3476 | *hˉ ade6-M216 ura4-D18 leu1-32 dcr1::natMX6* (pREP3X) | This study |
| BP3477 | *hˉ ade6-M26 ura4-D18 leu1-32 dcr1::natMX6* (pREP3X-*rnh1*^+^) | This study |
| BP3478 | *hˉ ade6-M26 ura4-D18 leu1-32 rnh201::hphMX6 tsn1::kanMX6* (pREP3X) | This study |
| BP3479 | *hˉ ade6-M26 ura4-D18 leu1-32 rnh201::hphMX6 tsn1::kanMX6* (pREP3X-*rnh1*^+^) | This study |
| BP3482 | *hˉ ade6-M26 ura4-D18 leu1-32 dcr1::natMX6* (pREP3X-*pac1*^+^) | This study |
| BP3483 | *hˉ ade6-M26 ura4-D18 leu1-32 dcr1::natMX6 tsn1::kanmx6* (pREP3X-*pac1*^+^) | This study |
| BP3484 | *hˉ ade6-M26 ura4-D18 leu1-32 rnh201::kanMX6 tsn1::natMX6* (pREP3X-*pac1*^+^) | This study |
| BP3488 | *hˉ ade6-M26 ura4-D18 leu1-32 dcr1::natMX6 tsn1::kanMX6* (pREP3X-Sp*tsn1*^+^) | This study |
| BP3489 | *hˉ ade6-M26 ura4-D18 leu1-32 dcr1::natMX6 tsn1::kanMX6* (pREP3X-Hs*TSN*^+^) | This study |
| BP3491 | *h- ade6-M26 leu1-32 ura4-D18 rnh201::kanMX6 tsn1::natMX6* (pREP3X-Sp*tsn1*^+^) | This study |
| BP3492 | *h- ade6-M26 leu1-32 ura4-D18 rnh201::kanMX6 tsn1::natMX6* (pREP3X-Hs*TSN*^+^) | This study |
| BP3493 | *h- ade6-M26 leu1-32 ura4-D18 nh201::kanMX6tsn1::natMX6* (pREP3X-*dcr1*^+^) | This study |
| BP3497 | *hˉ ade6-M26 ura4-D18 leu1-32 dcr1::natMX6 tsn1::kanMX6* (pREP3X-Sp*tsn1-E152A*) | This study |
| BP3499 | *hˉ ade6-M26 ura4-D18 leu1-32 dcr1::natMX6 tsn1::kanMX6* (pREP3X-Hs*TSN-E150A*) | This study |
| BP3510 | *hˉ ade6-M26 ura4-D18 leu1-32 dcr1::natMX6 tsn1::kanMX6* (pREP3X-*rnh201*^+^) | This study |
| BP3511 | *hˉ ade6-M26 ura4-D18 leu1-32 rnh1::kanMX6 rnh201::hphMX6* (pREP3X-*rnh201*^+^) | This study |
| BP3512 | *h- ade6-M26 leu1-32 ura4-D18 tsn1::kanMX6 dcr1::natMX6* (pREP3X-Hu*TSNAX*^+^) | This study |
| BP3513 | *h- ade6-M26 leu1-32 ura4-D18 tsn1::kanMX6 dcr1::natMX6* (pREP3X-Sp*tfx1*^+^) | This study |
| BP3518 | *hˉ ade6-M26 ura4-D18 leu1-32 dcr1::ura4*^+^ *rnh1::kanMX6* | This study |

**Reference**

1. Jain D, Hebden AK, Nakamura M, Miller KM, Cooper JP. HAATI survivors replace canonical telomeres with blocks of generic heterochromatin. Nature. 2010; 467: 223-227. https://doi.org/10.1038/nature09374 PMID: 20829796
