# Supplementary material for: Translin facilitates RNA polymerase II dissociation and suppresses genome instability during RNase H2- and Dicer-deficiency
Source: PLoS Genet. 2022 Jun 17;18(6):e1010267. doi: 10.1371/journal.pgen.1010267 (PMC9246224; doi:10.1371/journal.pgen.1010267)
Supplement: S2 Table — (DOCX) [file pgen.1010267.s002.docx]

**S2 Table.** Primers used in this study.

| Primer designation | Primer sequence (5'-3') |
| --- | --- |
|  |  |
| rDNA qPCR F | TTTCTAGGACCGCCGTAATG |
| rDNA qPCR R | TGCTTTCGCAGTAGTTCGTC |
| HIS.02 qPCR F | CTGGTGTGGGCACTTACTAT |
| HIS.02 qPCR R | ATGGATCTATTTGGGATGC |
| MET.06 qPCR F | TCCTGGGACCTACGGGTTAT |
| MET.06 qPCR R | AACGGATATAGGTTCAT |
| ARG.09 qPCR F | GGTTAAGGCGCTTGACTACG |
| ARG.09 qPCR R | ACATCCTTTTTGCACTCGAA |
| PRO.01 qPCR F | CACAATATCAACTGAGGCTTCG |
| PRO.01 qPCR R | AAATTTAAAGGCTTTGGGCTTC |
| VAL.01 qPCR F | ACAACCAACAGTCCCGTGTT |
| VAL.01 qPCR R | TGGTTCAAGTTCGCTATTGTTG |
| ASN.03 qPCR F | AAGCAAGAAGGTCGGGTAG |
| ASN.03 qPCR R | TGTGCGTTTGTCTATCCTTTGT |
| TYR.01 qPCR F | AACTCCTGATGGTGTAGTTGGT |
| TYR.01 qPCR R | TTTACCAGGTGGAAGCA |
| pac-1 cloning F* | ccgctcgagATGGGACGGTTTAAGAGGCA |
| pac-1 cloning R* | cgcggatccTTAACGGGCAAACTTAGAGTAATC |
| rnh1 cloning F* | cgcggatccATGGGTGGAAATAAGCGTGC |
| rnh1 cloning R* | cgcggatccTTACTCAGAAGCTCCTCGCC |
| rnh201 cloning F* | cgcggatccATGAAAGATGATCACGATGC |
| rnh201 cloning R* | cgcggatccCTAAAAATAAAACTCTGATC |
| dcr1 cloning F* | cgcggatccATGGATATTTCAAGTTTTCTACTTC |
| dcr1 cloning R* | cgcggatccTCAAGTCAAACTTTTAACTTTTCC |
| Sp Tsn1 cloning F* | cgcggatccATGAATAAATCAATATTTATTCAGCTA |
| Sp Tsn1 cloning R* | cgcggatccTTAAACCAATTTATGTATCCGAAG |
| Hs TSN cloning F* | cgcggatccATGTCTGTGAGCGAGATCTTCG |
| Hs TSN cloning R* | cgcggatccCTATTTTTCAACACAAGCTGCTG |
| Sp Tfx1 cloning F* | cgcggatccATGGAAGAGGAATTCCTCTCA |
| Sp Tfx1 cloning F* | cgcggatccTTATGTGGACCGTAATCGTTTC |
| Hs TSNAX cloning F* | cgcggatccATGAGCAACAAAGAAGGATCAG |
| HS TSNAX cloning R* | cgcggatccCTAAGAAATGCCCTCTTCTTG |

*Lower case = restriction site
