# Supplementary figures and images for: Translin facilitates RNA polymerase II dissociation and suppresses genome instability during RNase H2- and Dicer-deficiency
Source: PLoS Genet. 2022 Jun 17;18(6):e1010267. doi: 10.1371/journal.pgen.1010267 (PMC9246224; doi:10.1371/journal.pgen.1010267)

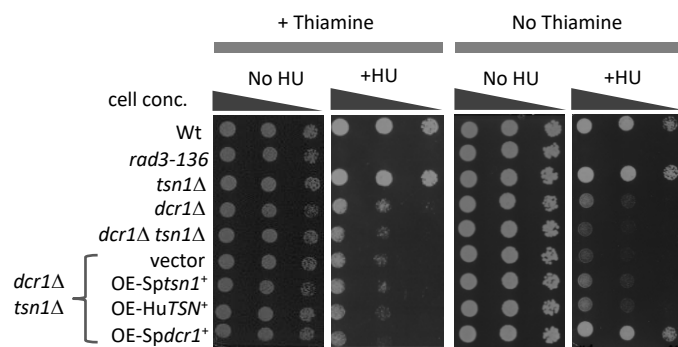

Supplement: S1 Fig — Appropriate strains were spotted onto EMM media with (left hand pair) and without (right hand pair) thiamine [which suppresses gene overexpression (OE) from pREP3X nmt promoter] containing no HU or 10 mM HU. In the presence of thiamine the overexpression of either S. pombe tsn1+ (Sptsn1+), S. pombe dcr1+ (Spdcr1+) or human TSN+ (HuTSN+) result in suppression of the elevated HU sensitivity of the S. pombe dcr1Δ tsn1Δ double mutant. (PDF) [file pgen.1010267.s003.pdf]

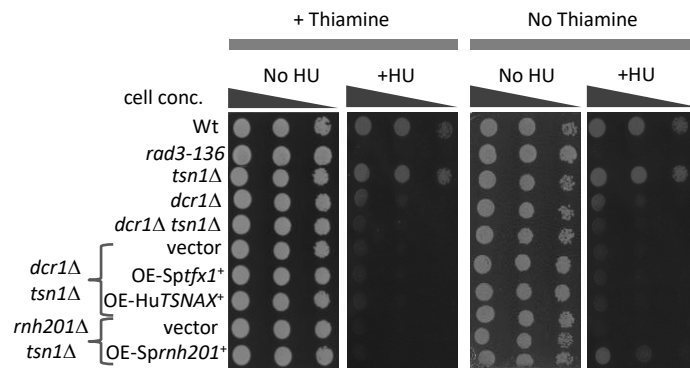

Supplement: S2 Fig — 10-fold serial dilutions of indicated strains were spotted onto EMM with thiamine (with and without HU; 10 mM) or without thiamine (with or without HU; 10 mM). OE-Sptfxfx—overexpressed S. pombe tsn1+; OE-HuTSNAX+—overexpressed human TSNAX+; OE-Sprnh1+—overexpressed S. pombe rnh1+. (PDF) [file pgen.1010267.s004.pdf]

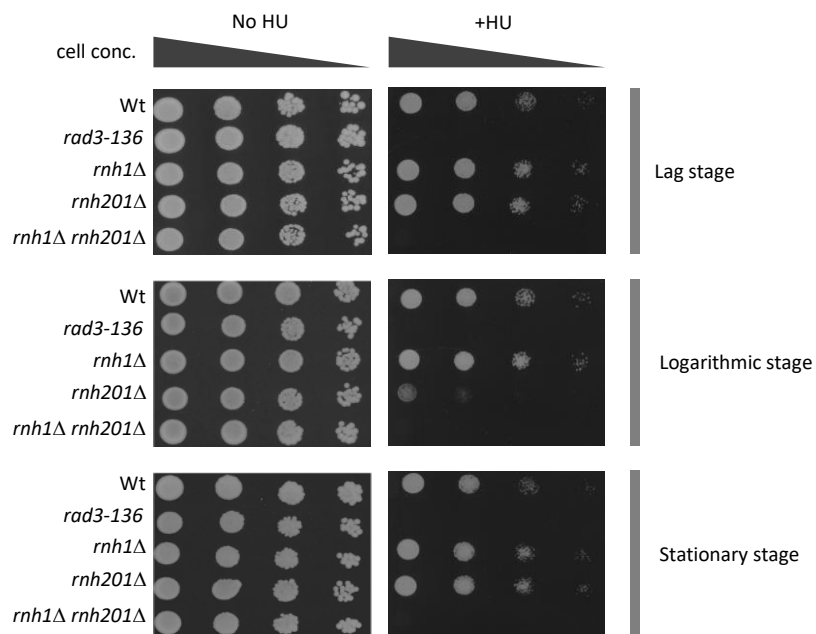

Supplement: S3 Fig — Appropriate S. pombe strains were spotted out onto YEA without (left hand set) or with 10 mM HU (right hand set). The same cultures were spotted out from the three distinct phases of proliferative expansion, lag (top row), logarithmic (middle row) or stationary (bottom row). Only logarithmically proliferating rnh201Δ single mutants exhibited HU sensitivity. rnh1Δ single mutants exhibited no HU sensitivity for all culture stages. (PDF) [file pgen.1010267.s005.pdf]

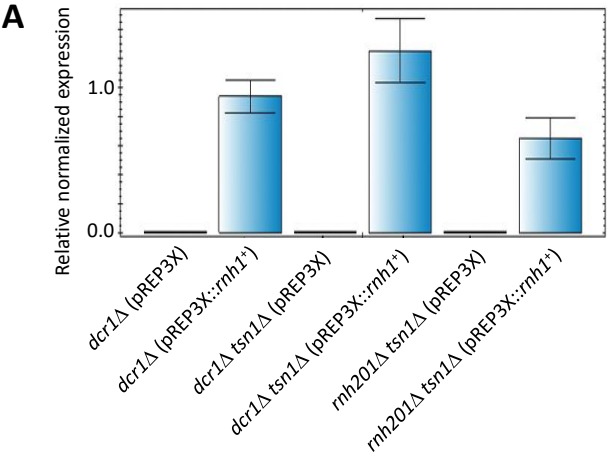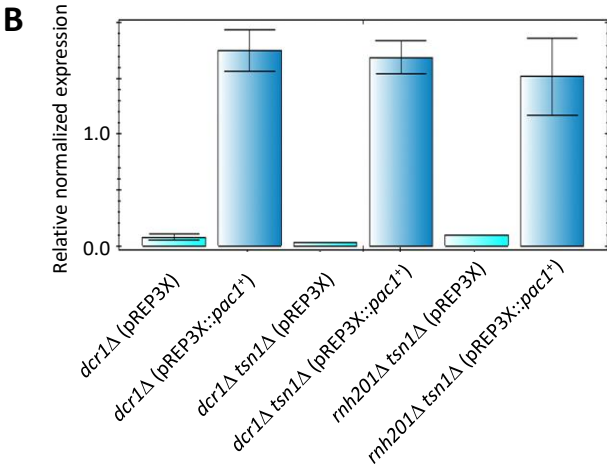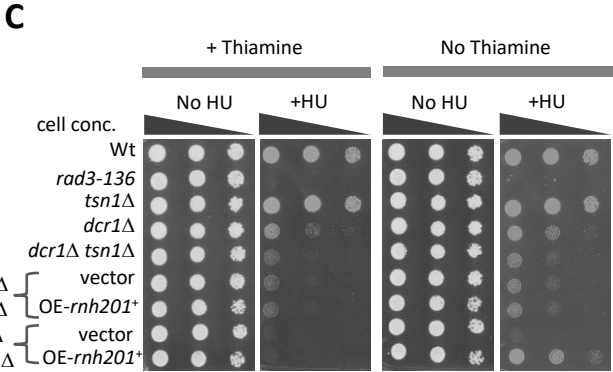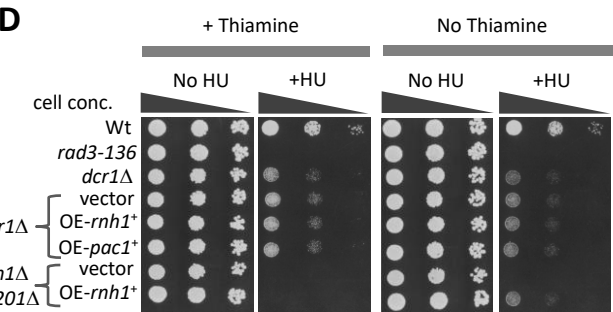

Supplement: S4 Fig — (A) RT-qPCR analysis of expression of rnh1+ in distinct strains. pREP3X is the empty vector control. Normalization was carried out against act1+ levels. Error bars represent standard deviation (triplicate biological repeats). (B) RT-qPCR analysis of expression of pac1+ in distinct strains. pREP3X is the empty vector control. Normalization was carried out against act1+ levels. Error bars represent standard deviation (triplicate biological repeats). (C) Overexpression of rnh201+ does not suppress requirement for Tsn1 to tolerate HU in the absence of Dcr1. OE = overexpression. In the presence of thiamine rnh201+ is not overexpressed from the vector (pREP3X); overexpression is triggered upon the removal of thiamine. Overexpression of rnh201+ in the absence of thiamine suppresses the HU sensitivity phenotype of the rnh1Δ rnh201Δ double mutant, which demonstrates the rnh201+ overexpression is active on these plates. (D) Overexpression of rnh1+ or pac1+ does not suppress requirement for Dcr1 to tolerate HU. OE = overexpression. In the presence of thiamine rnh1+ and pac1+ are not overexpressed from the vector (pREP3X). Overexpression of rnh1+ in the absence of thiamine suppresses the HU sensitivity phenotype of the rnh1Δ rnh201Δ double mutant, which demonstrates the rnh201+ overexpression is active on these plates. (PDF) [file pgen.1010267.s006.pdf]

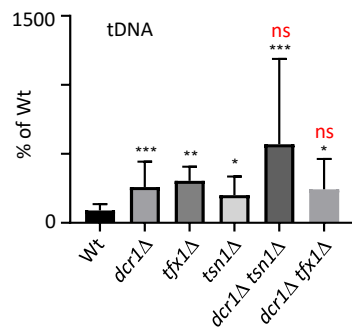

Supplement: S6 Fig — Quantification of DRIP for the tDNAHIS locus for the indicated strains. *P>0.05, **P>0.01, ***P>0.001, ns–not significant, from t-test in pairwise comparisons relative to relative to wild-type (black) or to dcr1Δ (red). Bars represent standard deviation. Values are obtained from a minimum of three independent biological repeats. (PDF) [file pgen.1010267.s008.pdf]

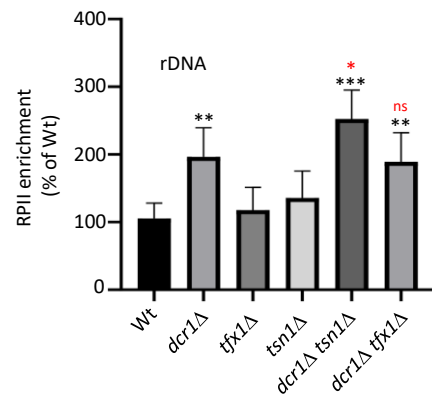

Supplement: S7 Fig — Quantification of RPII ChIP for the rDNA locus for the indicated strains. *P>0.05, **P>0.01, ***P>0.001, ns–not significant, relative to the wild-type (black) or to dcr1Δ (red). Bars represent standard deviation. Values are obtained from a minimum of three independent biological repeats. (PDF) [file pgen.1010267.s009.pdf]
