# Supplementary material for: Translin facilitates RNA polymerase II dissociation and suppresses genome instability during RNase H2- and Dicer-deficiency
Source: PLoS Genet. 2022 Jun 17;18(6):e1010267. doi: 10.1371/journal.pgen.1010267 (PMC9246224; doi:10.1371/journal.pgen.1010267)

A

```
Hu TSN     ---------------------------------MSVSEIFVELQGFLAAEQDIREEIRKVV 28
Sp Tsn1    ----------------------------------MNKSIFIQLQDQIDKEHSIREKLTAEV 27
Hu TSNAX   MSNKEGSGGFRKRKHDNFPHNQRREGKDVNSSSPVMLAFKSFQQELDARHDKYERLVKLS 60
Sp Tfx1    -----------------------------------MEEEFLSFKNFLQEDQDKREKIIRLS 26
                                                 * .::  :   :.  *.:

Hu TSN      QSLEQTAREILTLLQGVHQGAGFQDIPKRCLKAREHFGTVKTHLTSLKTKFPAEQYYRFH 88
Sp Tsn1    DLLDEKLRVLQLLLANCEQNLENQE---EILEAL---EIIKSKTRGLAELASNFPYYKYN 81
Hu TSNAX   RDITVESKRTIFLLHRITSAPDMED---ILTESEIKLDGVRQKIFQVAQELSGEDMHQFH 117
Sp Tfx1    REITIQSKRMIFLLHQTSSSDGFPL-PKDFDRTSIFEKKIHKELESLKRELAGLNADKFS 85
             :    :    **    .            .:      :: .   :          ::

                  E126 E129
Hu TSN      EHWRFVLQRLVFLAAFVVYLETET---------LVTREAVTEILGI-EPD----------- 128
Sp Tsn1    GVWDRSIQKVVYLYLLASWTGRLDKSLRPTYSLLSLSEVGQILQVPVFP----------- 130
Hu TSNAX   RAITTGLQEYVEAVSFQHFIKTRS---------LISMDEINKQLIFTTEDNGKENKTPSSD 169
Sp Tfx1    SACTHGLQEYVEAVTFKFWLQTGT---------LLSCKD---------------------- 115
                 :*. *    :  :             *:: .

                                D193 E197
Hu TSN      ----REKGFH--LDVEDYLSGVLILASELSRLSVNSVTAGDYSRPLHISTFINELDSGFR 182
Sp Tsn1    ----EESTFH--LSIEQYLHAVLSLCSELARQSVNSVISGNYHIPFEALNTIQKVHSSFQ 184
Hu TSNAX   AQDKQFGTWRLRVTPVDYLLGVADLTGELMRMCINSVGNGDIDTPFEVSQFLRQVYDGFS 229
Sp Tfx1    ------SSFRISINFIDYVLGVCDMTGEIMRFLVTNGSKFSVQQLTQQVKFLRGLHKNCS 169
                   ::  :   :*: .*  : .*: *  :..    .     .    :. : ..

Hu TSN      LLNL----KNDSLRKRYDGLKYDVKKVEEVVYDLSIRGFNKETAAAC----VEK------ 228
Sp Tsn1    VLSL----KNDSLRRHFDGLKYDLKRSEDVVYDLRIHKLV-------------------- 220
Hu TSNAX   FI---GNTGPYEVSKKLYTLKQSLAKVENACYALKVRGSEIPKHMLADVFSVKTEMIDQE 286
Sp Tfx1    EIEHLPSKVKSELQQKLSVMENSISKVEGICYSKILREADKRYLNLE----VDTATPPEE 225
            :         .: ::   :: .: : *   *   ::

Hu TSN      ------  228
Sp Tsn1    ------  220
Hu TSNAX   EGIS--  286
Sp Tfx1    KRLRST  225
```

B

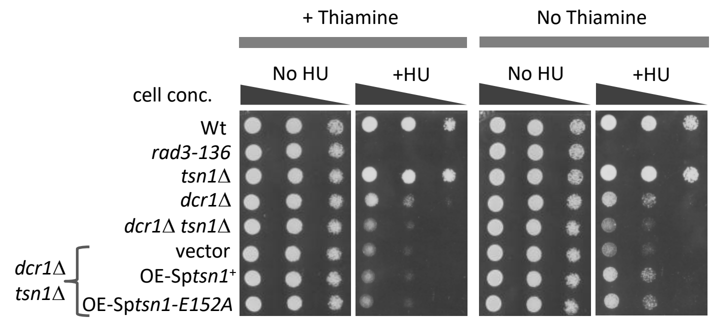

Supplement: S5 Fig — (A) Alignment of human and S. pombe Translin (Hu TSN and Sp Tsn1, respectively) and Trax (Hu TSNAX and Sp Tfx1, respectively) paralogues. Amino acids highlighted in blue are required for Trax RNase activity and are not conserved in Translin. The residue marked in red (E197) is an RNase catalytic residue for human TSNAX (Trax) which is the only RNase catalytic residue to be conserved in both species. Amino acid residue numerical designations refer to the human TSNAX sequence. (B) Mutation of the conserved RNase catalytic residue in S. pombe tsn1 does not disrupt genome stability function. Over expression (OE) of the S. pombe tsn1-E152A mutant allele can suppress the loss of tsn1 function to the same degree as the tsn1+ wild-type control. (PDF) [file pgen.1010267.s007.pdf]
